# Combined Drug Targeting of p53-dependent and -independent Pathways Depletes Myelofibrosis Hematopoietic Stem/Progenitor Cells

**DOI:** 10.1038/s41375-021-01446-4

**Published:** 2021-10-12

**Authors:** Min Lu, Lijuan Xia, Nada Elmansy, Cara Clementelli, Douglas Tremblay, Ronald Hoffman

**Affiliations:** Division of Hematology/Oncology, Tisch Cancer Institute and Department of Medicine, Myeloproliferative Neoplasm-Research Consortium, Icahn School of Medicine at Mount Sinai, New York, New York 10029 USA

**Keywords:** Myeloproliferative disease, Apoptosis

## Abstract

Current therapy for myelofibrosis (MF) results in a limited prolongation of patient survival. In order to improve treatment outcomes, we developed a strategy to effectively deplete MF hematopoietic stem/progenitor cells (HSPCs). In the present study, an imipridone, ONC201, was combined with RG7112, an antagonist of MDM2, a p53 negative regulator, to activate downstream events of the p53 and TNF-related apoptosis-inducing ligand (TRAIL)/death receptor (DR) pathways. As compared to treatment with the individual drugs, the combination of ONC201 and RG7112 promoted greater degrees of apoptosis of MF CD34^+^ cells through activation of both p53-dependent and -independent pathways. Importantly, treatment with ONC201-RG7112 not only decreased the number of *JAK2V617F*^+^ and calreticulin mutated colonies assayed from MF CD34^+^ cells, but allowed for the persistence or appearance of JAK2 wild type colonies. Treatment with ONC201 combined with RG7112 could be a potentially effective strategy for treating MF patients.

## Introduction

Myelofibrosis (MF), a myeloproliferative neoplasm (MPN), arises at the level of hematopoietic stem progenitor cells (HSPCs) due to a series of mutational events that result in activation of JAK/STAT signaling [1, 2]. The driver mutations associated with MF involve *JAK2*, calreticulin (CALR), and the thrombopoietin receptor, *MPL* [1, 3–5]. Additional mutations can accompany these driver mutations including epigenetic modifiers (*DNMT3A*, *TET2*, and *ASXL1*), splicing factors (*SF3B1*, *SRSF2*, and *U2AF1*), metabolic enzymes (*IDH1*, *IDH2*), and tumor suppressors (*TP53*) which are associated with an inferior survival of MF patients [6–8]. JAK/STAT signaling has served as the primary target for MPN specific drug development and has led to the approval of two small-molecule JAK2 inhibitors, ruxolitinib and fedratinib [2, 9, 10]. JAK2 inhibitor therapy for patients with advanced forms of MF results in a reduction in the degree of splenomegaly and improvement in the systemic symptoms but does not prevent disease progression and only modestly prolongs overall survival [11–14].

Increased expression of MDM2, a negative regulator of p53, has been observed in MPN CD34^+^ HSPCs [15]. Nakatake et al demonstrated that *JAK2V617F* alters p53 responses to DNA damage by up-regulating the La-antigen which increases MDM2 protein translation [16]. We have shown that MDM2 antagonists termed nutlins can selectively eliminate MPN CD34^+^ cells by activating the p53 pathway [17–19]. We have also reported a phase 1 trial of the oral nutlin, RG7388 (idasanutlin), in polycythemia vera (PV) patients intolerant or refractory to hydroxyurea or interferon therapy, which resulted in hematological responses, reduction in the degree of splenomegaly, an improvement in symptoms and a reduction in the *JAK2V617F* variant allele frequency (VAF) [20]. Additional trials of MDM2 antagonists in MF patients have led to reductions in spleen size, improvement of systemic symptoms, and reduction in the VAF of MPN driver mutations (NCT04097821, NCT04485260, NCT04640532). Resistance to MDM2 inhibitors has been evaluated in solid tumor cell lines and has been attributed to either the emergence of de novo *TP53* mutations or the selection of *TP53* mutated clones [21, 22]. We have recently observed that nutlin treatment of PV patients was associated with the transient increase in the VAF of *TP53* mutations that were present prior to initiation of nutlin therapy. Fortunately, the appearance of these *TP53* mutations diminished with discontinuation of nutlin therapy and was not associated with drug resistance or progression to MF or MPN-blast phase (BP) [23].

Since the chronic administration of idasanutlin in PV, leukemia, and solid tumor patients has been limited due to gastrointestinal toxicity [20, 24–26], we have searched for alternative drugs which target different pathways to partner with a nutlin with the goal of developing a more effective and tolerable stem cell depleting therapy for MF patients.

Oral imipridone, ONC201, triggers apoptosis by activating the extrinsic apoptosis pathway [27–30]. It transcriptionally induces TNF-related apoptosis-inducing ligand (*TRAIL*) which binds to members of the TNF-alpha receptor super-family, including the death receptors 4 and 5 (DR4 and DR5), thereby inducing apoptosis through activation of caspase-8 [31]. Recent studies have shown that ONC201 increases TRAIL, DR4, and DR5 at both the transcriptional and translational levels by inactivating the AKT/ERK pathways and leading to upregulation and translocation of Foxo3a into the nucleus where it binds to the TRAIL promoter to upregulate gene transcription [30, 32, 33]. The actions of ONC201 on malignant cells are known to be independent of p53 [32, 34].

ONC201 has also been shown to be a potent allosteric agonist of the ATP-dependent mitochondrial caseinolytic protease P (ClpP), which regulates oxidative phosphorylation by controlling the degradation of the respiratory chain components and triggering the mitochondrial unfolded protein response [35, 36]. Hyper-activation of ClpP by ONC201 increases mitochondrial proteolysis and leads to mitochondrial dysfunction, impaired oxidative phosphorylation, and death of acute myeloid leukemia cells [37–39]. The effects of ONC201 on AML cells are thought to be through its actions on ClpP rather than the TRAIL/DR pathway. Ishizawa and Geer have shown that ONC201 induces the atypical integrated stress response (ISR) in various types of malignant cells by activating transcription factor 4 (ATF4) [34, 37]. Deleting ATF4, or inhibiting events upstream of the ISR, blocks the induction of ATF4-target genes thereby limiting the effects of ONC201. Whether ONC201 affects MF HSPC ClpP has not been previously explored.

TP53 is also known to induce the expression of TRAIL, DR4, and DR5 [40], we then hypothesized that the effects of ONC201 on the extrinsic apoptosis pathway and mitochondrial function would be complementary with the consequences of upregulation of p53 achieved. We evaluated if a combination of a nutlin and ONC201 might prove to be an effective therapeutic strategy to selectively induce apoptosis of malignant MF CD34^+^ cells.

## Materials and methods

### Specimen collection and cell preparation

Primary samples were collected from 21 individual MF patients after written informed consent was obtained according to guidelines established by the Institutional Review Board of the Icahn School of Medicine at Mount Sinai (ISMMS). The study was conducted in accordance with the Principles of the Declaration of Helsinki. All patients met the World Health Organization criteria for the diagnosis of MF [41, 42]. Patient characteristics are provided in Supplemental Table 1. Normal donor (ND) human bone marrow (BM) was purchased from AllCells (Emeryville, CA). The experiments in which the CD34^+^ cells from each of the patients were studied are itemized in Supplemental Table 2.

### Flow cytometry assays

Cells were collected and washed in MACS buffer twice and then stained with the designated antibodies directly for at least 15 min prior to further analysis. Data were acquired on a FACS Calibur analyzer (Becton Dickinson, Franklin Lakes, NJ).

### Isolation of RNA and qRT-PCR

Total RNA was extracted from treated CD34^+^ cells using an RNeasy kit (QIAGEN, Valencia, CA). Complementary DNA was reverse transcribed using the EcoDry Premix kit (Clontech Laboratories, Mountain View, C.A.). The targeting transcripts were evaluated by quantitative reverse-transcription (qRT)-PCR.

### Activation of *TP53* transcript in CD34^+^ cells

MF CD34^+^ cells were transiently transfected with either control CRISPR/dCas9 activation plasmid or p53 CRISPR/Cas9 activation plasmid using Amaxa Human CD34^+^ cell nucleofector kit (Lonza, Alpharetta, GA). Control CRISPR activation plasmid and human p53 CRISPR activation plasmid were purchased from Santa Cruz Biotechnology (Dallas, TX).

### Western blot analysis

MF CD34^+^ cells were harvested after being treated with either each drug alone or in combination, and whole-cell protein extracts were prepared with RIPA lysis buffer (Boston BioProducts, Worcester, MA) containing a protease inhibitor cocktail (Thermo Fisher Scientific) for western blotting.

### Hematopoietic progenitor cell proliferation assays

HSPCs were assayed in semisolid media as described previously [18]. The hematopoietic colonies were enumerated after 14 days of incubation with or without drugs. The individual colonies were plucked and genotyped for JAK2V617F [15].

### Nested allele-specific polymerase chain reaction for JAK2V617F

Genomic DNA was isolated from randomly plucked colonies and JAK2V617F was detected by using a nested allele-specific PCR.

### Statistical analysis

Results were reported as the mean ± standard deviation. A two-sided Student’s *t*-test was used for quantitative assays. For two-group comparisons, non-parametric Wilcoxon Rank Sum Tests were used to assess differences in distributions of quantitative variables. Statistical significance was established at *P* < 0.05.

Additional details regarding reagents, patient samples, experimental methods are found in the online Supplemental Materials.

## Results

### MF CD34^+^ cells express different levels of MDM2, TRAIL, and DRs as compared to ND CD34^+^ cells

**Fig. 1:**
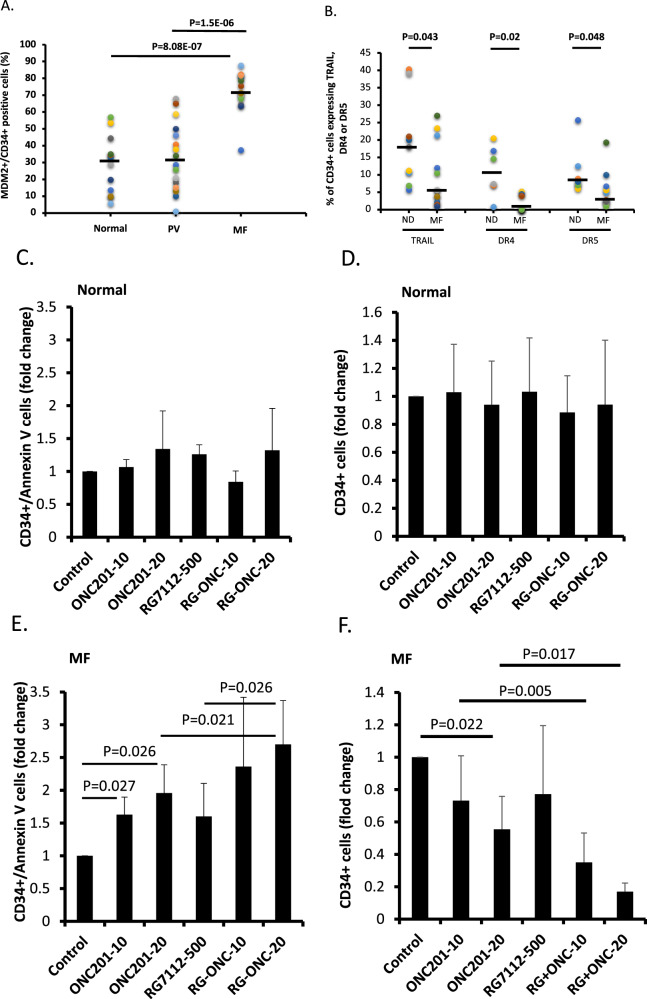
Comparison of protein levels of MDM2, TRAIL and death receptors in MF and ND CD34^+^ cells and the effects of treatment with ONC201 and RG7112 on apoptosis of MF and ND CD34^+^ cells. **A** Flow cytometric (FC) analysis data showed a greater percentage of MF CD34^+^ cells expressed MDM2 as compared to normal CD34^+^ cells (*P* = 8.08E^−07^) and PV CD34^+^ cells (*p* = 1.5E^−06^ normal *n* = 12, PV *n* = 16, MF *n* = 13); (**B**) FC data showed a lower percentage of MF CD34^+^ cells expressed TRAIL (*n* = 12), DR4 (*n* = 8) and DR5 (*n* = 10) than ND CD34^+^ cells (ND *n* = 9, *n* = 6 and *n* = 8) (*p* = 0.043, *p* = 0.02 and *p* = 0.048, respectively). In figure (**A**) and (**B**), statistical analyses were performed by non-parametric Wilcoxon Rank Sum Tests. Each dot representative the data from an individual case and short black line indicates the mean value in each group; (**C**) Neither treatment with ONC201 and RG7112 alone nor in combination induced apoptosis of ND CD34^+^ cells (*n* = 4); (**D**) Treatment with ONC201 and RG7112 alone and in combination did not significantly decrease the number of ND CD34^+^ cells (*n* = 4); (**E**) Treatment with ONC201 and RG7112 alone or in combination induced apoptosis of MF CD34^+^ cells (*n* = 6) (*p* = 0.027 (con vs. ONC201 10uM), *p* = 0.026 (con vs. ONC201 20 uM); *p* = 0.026 (ONC201 vs. RG7112-ONC201) and *p* = 0.03 (RG7112 vs. RG7112-ONC201)). **F** Treatment with ONC201 and RG7112 alone and in combination significantly decreased the number of MF CD34^+^ cells (*n* = 6), the combination of the two drugs led to a significantly greater reduction of MF CD34^+^ cell numbers than that observed with ONC201 alone (*p* = 0.005 and *p* = 0.017, respectively). Data are mean ± S.D. Analyses were performed by *t*-test.

We first demonstrated that a higher percentage of MF CD34^+^ cells were MDM2^+^ than either ND or PV CD34^+^ cells (Fig. 1A). This increased expression of MDM2 was not different in MF patients with CALR mutations and with *JAK2V617F* (Suppl. Fig. 1). The data also showed that the percentage of TRAIL^+^, DR4^+^ or DR5^+^ cells was significantly decreased in MF CD34^+^ cells as compared to ND CD34^+^ cells (Fig. 1B, Suppl. Fig. 2).

### Treatment with ONC201 and RG7112 induces apoptosis of MF CD34^+^ cells but not ND CD34^+^ cells

Treatment with ONC201 or RG7112 alone or in combination for two days did not induce apoptosis of ND CD34^+^ cells (Fig. 1C, Suppl. Figure 3A), which is reflected by an absence of change in the number of ND CD34^+^ cells in all treatment groups (Fig. 1D). By contrast, treatment with ONC201 increased apoptosis of MF CD34^+^ cells in a dose-dependent fashion. Combination treatment with ONC201 + RG7112 induced apoptosis of MF CD34^+^ cells to a statistically greater degree than either drug alone (Fig. 1E, Suppl. Figure 3B). The CD34^+^ cell numbers were decreased following treatment with ONC201. The combination of the two drugs led to a significantly greater reduction of MF CD34^+^ cell numbers than that observed with ONC201 alone (Fig. 1F).

### Treatment with ONC201 and RG7112 increased expression of TRAIL and DR5 by MF CD34^+^ cells but not by ND CD34^+^ cells

**Fig. 2:**
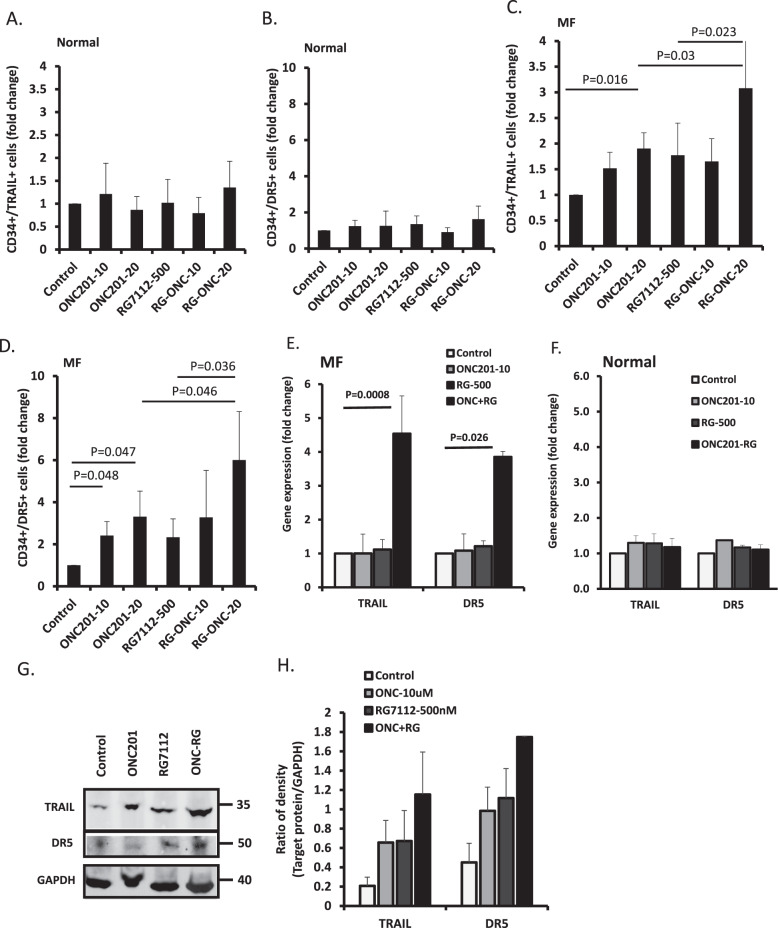
Treatment with ONC201 + RG7112 increases the percentage of MF CD34^+^ cells but not ND CD34^+^ cells that express TRAIL and DR5. **A** Treatment with ONC201 and RG7112 alone and in combination did not increase the percentage of normal CD34^+^/TRAIL^+^ cells (*n* = 4); **B** Treatment with ONC201 and RG7112 alone and in combination increased the percentage of MF CD34^+^/TRAIL^+^ cells (*n* = 6) (*p* = 0.015, con vs ONC201 20 uM; *p* = 0.03 as comb vs. ONC and *p* = 0.023 as comb vs RG); **C** Treatment with ONC201 and RG7112 alone and in combination did not increase the percentage of ND CD34^+^/DR5^+^ cells (*n* = 4); **D** Treatment with ONC201 and RG7112 alone and in combination increased the percentage of MF CD34^+^/DR5^+^ cells (*n* = 6); **E**, **F** A combination of low doses of RG7112 and ONC201 increased TRAIL and DR5 transcript levels in MF CD34^+^ cells (*n* = 3) but not ND CD34^+^ cells (*n* = 3); **G** Representative western blot analysis for TRAIL and DR5 in MF CD34^+^ cells performed with GAPDH as a loading control (repeated with two different individual MF cases); **H** The graph indicated the ratio of density (TRAIL/GAPDH and DR5/GAPDH) performed with Image J software based on western blot analysis, 3 individual MF cases were performed for TRAIL and 2 MF cases were performed for DR5. Data are mean ± S.D. Analyses were performed by *t*-test.

Treatment of ND CD34^+^ cells with ONC201 or RG7112 alone, or in combination for two days did not increase the percentage of TRAIL^+^/CD34^+^ and DR5^+^/CD34^+^ cells (Fig. 2A, B, Suppl. Fig. 4A and B). By contrast, treatment with either ONC201 or RG7112 alone increased the percentage of MF TRAIL^+^/CD34^+^ and DR5^+^/CD34^+^ cells, combination treatment with ONC201 + RG7112 further increased the MF TRAIL^+^/CD34^+^ and DR5^+^/CD34^+^ cells than that observed with either drug alone (Fig. 2C, D, Suppl. Fig. 4C and D).

Furthermore, we tested TRAIL and DR5 transcripts levels in ND and MF CD34^+^ cells after treatment with each drug alone or in combination for 16 h. Although treatment of both ND and MF CD34^+^ cells with ONC201 or RG7112 alone at that time point doses did not increase TRAIL or DR5 transcripts levels, combination treatment synergistically increased transcripts levels of TRAIL and DR5 in MF CD34^+^ cells but not ND CD34^+^ cells (Fig. 2E, F). Western blotting showed that treatment with ONC201 and RG7112 alone for two days increased TRAIL and DR5 protein levels while a combination of ONC201 and RG7112 further increased TRAIL and DR5 protein levels (Fig. 2G, H). These data indicate that the combination of ONC201 and RG7112 affects both the transcription and translation of these components of the extrusive apoptosis pathway in MF but not ND CD34^+^ cells.

### Treatment with ONC201 and RG7112 induced apoptosis of MF CD34^+^ cells by activating both the death signaling pathway and p53 pathway

**Fig. 3:**
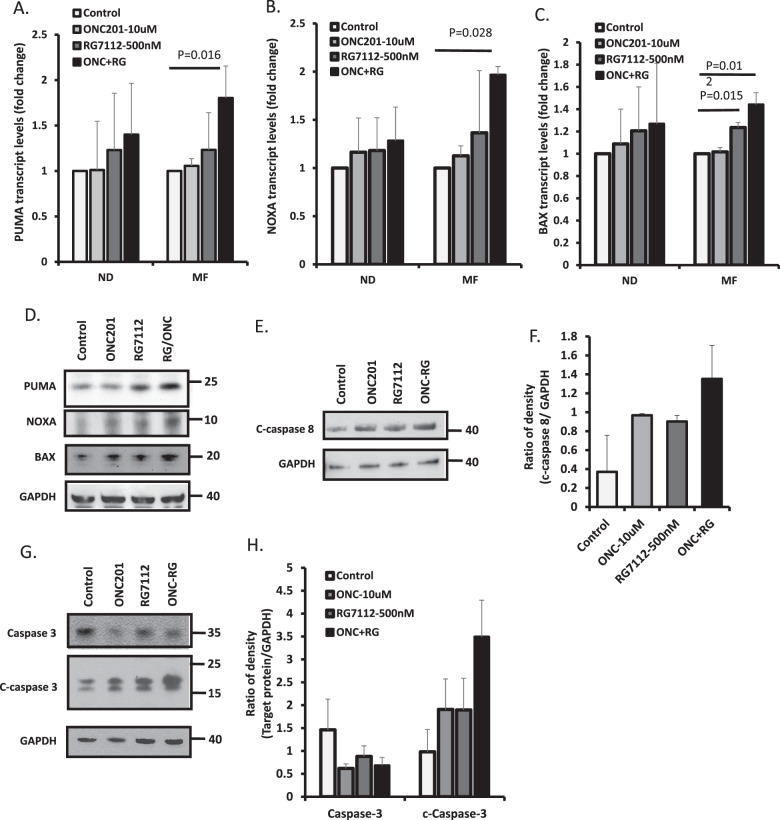
Treatment with ONC201 and RG7112 induced apoptosis of MF CD34^+^ cells by activating both death signaling pathway and p53 pathway. **A** Combination treatment with ONC201 and RG7112 increased PUMA transcript levels in MF CD34^+^ cells (*n* = 4) but not ND CD34^+^ cells (*n* = 3); **B** Combination treatment with ONC201 and RG7112 increased NOXA transcript levels in MF CD34^+^ cells (*n* = 4) but not ND CD34^+^ cells (n = 3); **C** Treatment with RG7112 alone or ONC201 + RG7112 increased BAX transcript levels in MF CD34^+^ cells (*n* = 4) but not ND CD34^+^ cells (*n* = 3); **D** Representative western blot analysis of MF CD34^+^ cells for PUMA, NOXA and BAX performed with GAPDH as a loading control (repeated with two different individual MF cases). **E** Representative western blot analysis of MF CD34^+^ cells for cleaved caspase-8 was performed with GAPDH as a loading control. **F** The graph indicated the ratio of density (c-caspase-8/GAPDH) performed with Image J software based on western blot analysis, 3 individual MF cases were performed; **G** Representative western blot analysis of MF CD34^+^ cells for caspase 3 and cleaved caspase 3 performed with GAPDH as a loading control; **H** The graph indicated the ratio of density (caspase-3/GAPDH and cleaved-caspase-3/GAPDH) performed with Image J software based on western blot analysis, 3 individual MF cases were performed. Data are mean ± S.D. Analyses were performed by *t*-test.

Activation of p53 by administration of RG7112 upregulates downstream pro-apoptosis genes, such as NOXA, PUMA, and BAX [43]. We then addressed the synergistic effects of treatment with ONC201 + RG7112 on activation of p53 to induce apoptosis. As one can see in Fig. 3, treatment of MF CD34^+^ cells with ONC201 alone did not increased NOXA, PUMA, and BAX transcript levels, treatment with RG7112 alone slightly increased the transcript levels of these three genes (Fig. 3A–C). While, the combination of ONC201 + RG7112 significantly increased transcript levels of NOXA, PUMA, and BAX (Fig. 3A–C). Western blotting confirmed that the proteins levels of these pro-apoptosis genes were increased (Fig. 3D). By contrast, treatment with ONC201 and RG7112 alone or in combination did not increase NOXA, PUMA, and BAX genes expression in ND CD34^+^ cells (Fig. 3A–C).

Caspase-3 activation promotes apoptosis due to activation of both the intrinsic (p53 pathway) and extrinsic (death ligand/caspase-8) apoptosis pathways [44]. As we can see in Fig. 3, treatment with either ONC201 or RG7112 increased the levels of cleaved-caspase-8, combination treatment further increase the levels of c-caspase-8 (Fig. 3E). furthermore, ONC201 and RG7112 alone increased cleaved-caspase-3 levels and decreased non-activated caspase 3, the combination of ONC201 + RG7112 doubled the levels of cleaved-caspase-3 as compared to each drug alone (Fig. 3F). These data indicate that combination treatment with ONC201 and RG7112 promote apoptosis of MF cells but not normal cells by activating both p53 and death signaling pathways.

### Treatment with ONC201 alone enhanced the effects of upregulated p53 on MF CD34^+^ cells but not ND CD34^+^ cells

**Fig. 4:**
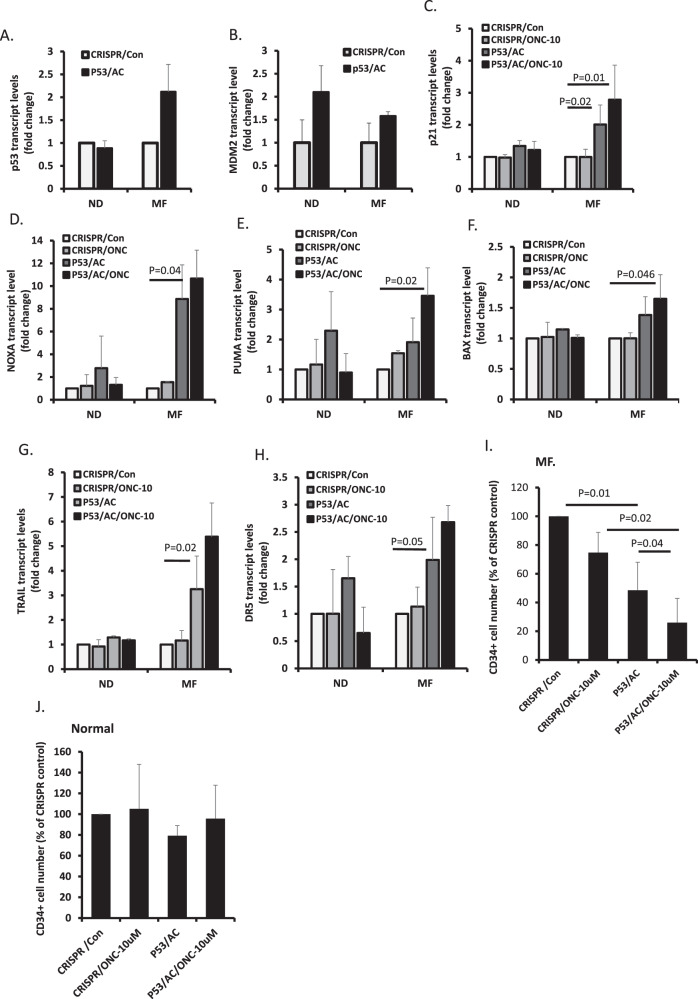
Treatment with ONC201 alone enhances the effects of upregulated p53 on MF CD34^+^ cells but not on ND CD34^+^ cells. Both ND and MF CD34^+^ cells were transfected with human p53 CRISPR activation plasmid to activate *TP53* transcription. CRISPR/Cas9 Activation Plasmids are a synergistic activation mediator (SAM) transcription activation system designed to specifically upregulate gene expression. **A** *TP53* transcript levels in ND and MF CD34^+^ cells transfected with control CRISPR activation plasmid and p53 activation plasmid for 3 days (ND *n* = 3, MF *n* = 4). **B** Transcript levels of MDM2 in ND and MF CD34^+^ cells transfected with control CRISPR activation plasmid and p53 active plasmid (ND *n* = 3, MF *n* = 4). **C** Transcript levels of p21 were increased in MF CD34^+^ cells transfected with p53 active plasmid but not ND CD34 + cells transfected with p53 active plasmid. The addition of ONC201 further increased p21 transcript levels in MF CD34^+^ cells with increased expression of *TP53* but not in the ND CD34^+^ cells nor MF CD34^+^ cells transfected with control CRISPR activation plasmid (ND *n* = 3, MF *n* = 4). **D** Transcript levels of NOXA were increased in ND CD34^+^ cells transfected with p53 active plasmid, but NOXA levels were increased more higher in MF CD34^+^ cells than ND CD34^+^ cells. The addition of ONC201 further increased NOXA transcript levels in MF CD34^+^ cells with increased expression of *TP53* but not in the ND CD34^+^ cells nor MF CD34^+^ cells transfected with control CRISPR activation plasmid (ND *n* = 3, MF *n* = 4). **E** and **F** Treatment with ONC201 increased PUMA and BAX expression in MF CD34^+^ cells with higher expression of *TP53* but not in the ND CD34^+^ cells nor cells transfected with the control plasmid (ND *n* = 3, MF *n* = 4). **G** and **H** MF CD34^+^ cells transfected with p53 activation plasmid expressed higher levels of both TRAIL and DR5. Treatment with ONC201 further increased TRAIL and DR5 expression with higher expression of *TP53* but not in the ND CD34^+^ cells nor cells transfected with the control plasmid (ND *n* = 3, MF *n* = 4). **I** CD34^+^ cell numbers were decreased after transfection with p53 activation plasmid, and were then further significantly reduced with the addition of ONC201 (*n* = 4). **J** Neither transfection with the p53 activation plasmid nor treatment with ONC201 decreased the numbers of ND CD34^+^ cell (*n* = 3). Data are mean ± S.D. Analyses were performed by *t*-test.

**Fig. 5:**
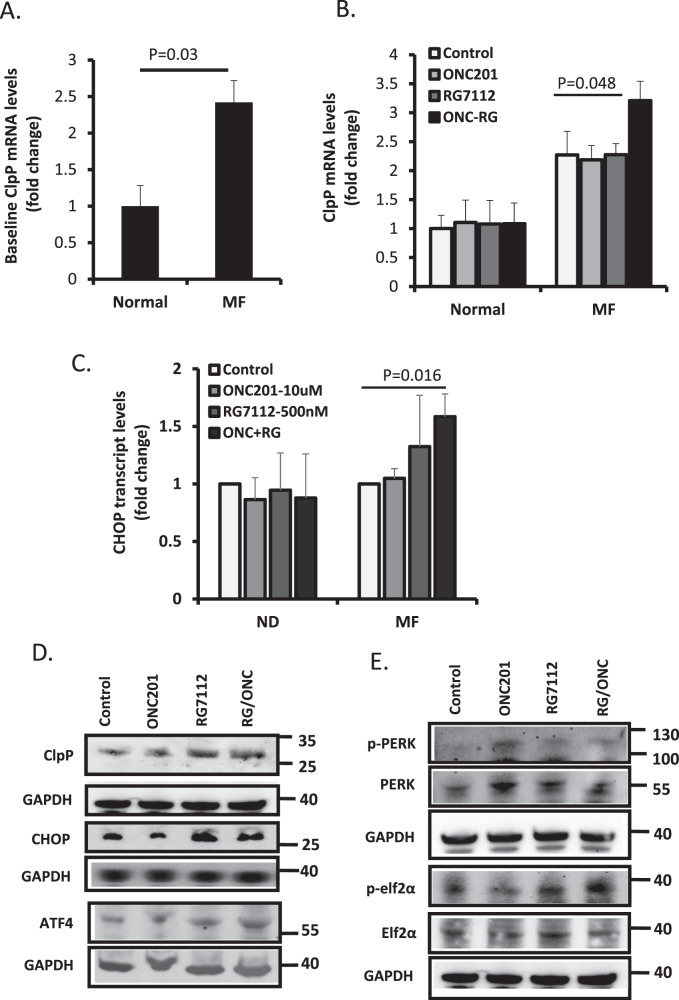
Treatment of MF CD34^+^ cells with ONC201 and RG7112 led to an increased stress response mediated through the endoplasmic reticulum and-mitochondria. **A** qRT-PCR showed that MF CD34^+^ cells were characterized by increased transcript levels of ClpP as compared to normal CD34^+^ cells (*p* = 0.03, normal *n* = 4, MF *n* = 6); **B** Treatment with ONC201 and RG7112 alone did not increase ClpP transcript levels of MF or ND CD34 + cells; combination treatment with ONC201 and RG7112 did further increased ClpP transcript levels in MF CD34^+^ cells but not ND CD34^+^ cells (ND *n* = 3, MF *n* = 4); **C** Combination treatment with ONC201 and RG7112 increased CHOP transcript levels in MF CD34^+^ cells but not ND CD34 + cells (ND *n* = 3, MF *n* = 4); **D** Representative western blot analysis of MF CD34^+^ cells for ClpP, CHOP, and ATF4 after treatment with OCN201 and RG7112 alone or in combination performed with GAPDH as loading control; **E** western blot analysis of MF CD34^+^ cells for p-PERK, PERK, p-ElF2α and ElF2α after treated with ONC201 and RG7112 alone or in combination performed with GAPDH as a loading control. Data are mean ± S.D. Analyses were performed by *t*-test.

In order to further confirm the additive effects of ONC201 on activation of p53, we transfected both ND and MF CD34^+^ cells with human p53 CRISPR activation plasmid to activate *TP53* transcription. The transfection efficiency for both ND and MF CD34^+^ cells was around 40% (Suppl. Fig. 5). *TP53* transcript levels were doubled in MF CD34^+^ cells transfected with p53 CRISPR activation plasmid when compared to cells transfected with control CRISPR activation plasmid after 3 days but not increased in ND CD34^+^ cells (Fig. 4A), while MDM2 transcript levels were increased to a higher level in ND CD34^+^ cells than that observed in MF CD34^+^ cells (Fig. 4B). This observed upregulation of MDM2 may blunt the effects of *TP53* on the ND CD34^+^ cells. Increased *TP53* expression in MF CD34^+^ cells was followed by its downstream target genes such as p21, PUMA, NOXA, and BAX expressing, the levels of these genes were further increased with the addition of ONC201 (Fig. 4C–E). Our results also showed that MF CD34^+^ cells with upregulated p53 transcription expressed higher levels of both TRAIL and DR5, and treatment with ONC201 further increased TRAIL and DR5 expression in these MF CD34^+^ cells (Fig. 4F, G). Although one can see the p53 downstream target genes p21, PUMA, and NOXA as well as TRAIL and DR5 were increased to varying degrees in ND CD34 ^+^ cells transfected with p53 activation plasmid, treatment with ONC201 did not further increase levels of these genes (Fig. 5C–H). Importantly, MF CD34^+^ cell numbers were significantly decreased after transfection with p53 activation plasmid, and were further decreased to a greater degree with the addition of ONC201 (Fig. 4I) but not decreased ND CD34 + cells (Fig. 4J).

### Treatment of MF CD34^+^ cells with ONC201 and RG7112 led to increased stress response through endoplasmic reticulum (ER) and mitochondria

To address whether ONC201 affects MF CD34^+^ cells by also altering ClpP, we evaluated the effects of treatment with ONC201 + RG7112 on transcript and protein levels of this mitochondrial protease. MF CD34^+^ cells were characterized by increased transcript levels of ClpP as compared to normal CD34^+^ cells (Fig. 5A). Although treatment with ONC201 and RG7112 alone for 16 h did not further increase ClpP transcript levels, combination treatment did increase ClpP transcript levels (Fig. 5B). In addition, combination treatment increased expression of C/EBP homologous protein (CHOP) transcript levels in MF CD34^+^ cells but not ND CD34^+^ cells (Fig. 5C). The data were then confirmed at the protein level by western blotting analysis (Fig. 5D). The ATF4, a transcription factor upstream of CHOP and downstream of ClpP, was modestly increased by treatment with ONC201 or RG7112 alone, yet the combination of ONC201 + RG7112 further increase ATF4 protein levels (Fig. 5D). CHOP is a multifunctional transcription factor that is upregulated in response to a wide variety of stresses [45]. The ATF4/CHOP-mediated integrated stress response leads to TRAIL/ DR5 activation [29]. Although we did not observe the clear results after treatment with ONC201 and RG7112 alone or ONC201 + RG7112 on the levels of phosphorylated protein kinase RNA-like endoplasmic reticulum kinase (PERK), RG7112 and ONC201 + RG7112 increased eukaryotic initiation factor 2 alpha (eIF2α) in MF CD34 + cells (Fig. 5E). The PERK/eIF2α/ATF4/CHOP signaling pathway plays an important roles in mitochondria and ER stress responses [46]. Due to the limited numbers of CD34^+^ cells present in the small amounts of blood, we were able to obtain from MF patients who were frequently anemic, insufficient cells to further study the effects of these drugs on oxidative phosphorylation were available.

### Treatment with ONC201 and RG7112 not only decreased colony numbers formed by MF progenitor cells but also reduced numbers of *JAK2V617F*^+^ HSPC

**Fig. 6:**
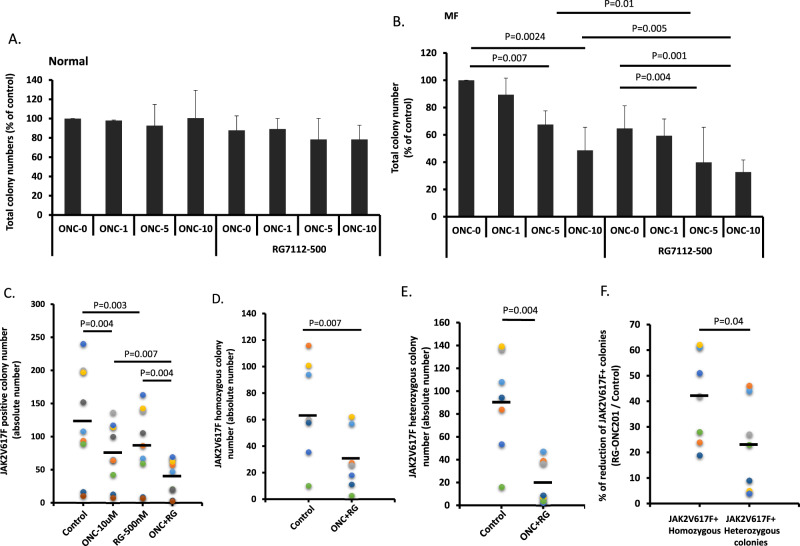
Treatment with ONC201-RG7112 decreases the total number and the number of JAK2V617F^+^ colonies formed by MF progenitor cells. **A**Treatment with ONC201 and RG7112 alone or in combination does not decrease colony formation by NDCD34^+^ cells (*n* = 6); **B** Treatment with ONC201 alone decreased total hematopoietic colonies generated by MF CD34^+^ cells in a dose-dependent fashion. Combination treatment withONC201and RG7112 more effectively decreased colony numbers generated by MF CD34^+^ cells (*n* = 16) (ONC201 vs. ONC-RG7112: *p* = 0.005 and RG7112 vs ONC-RG7112: *p* = 0.001, respectively); In (**A**) and (**B**), the experiment represents the total number of cases in both normal or MF that are represented together as a single data point with ranges of response. **C** Effects of treatment with ONC201 and RG7112 alone or in combination on the absolute numbers of hematopoietic colonies with JAK2V617F^+^ genotype. **D** Combination treatment with ONC201 and RG7112 decreased the absolute numbers of JAK2V617F^+^ homozygous hematopoietic colonies; **E** Combination treatment with ONC201 and RG7112 decreased the absolute numbers of assayable JAK2V617F^+^ heterozygous hematopoietic colonies; **F** Combination treatment with ONC201 and RG7112 more effectively decreased the numbers of assayable JAK2V616F^+^ heterozygous rather than homozygous hematopoietic colonies (*p* = 0.04). In figure **C**–**D**, **E**–**F**, each dot representative the data from an individual case, and a short black line indicates the mean value in each group. Analyses were performed by non-parametric Wilcoxon Rank Sum Tests.

We next evaluated whether treatment with ONC201 or RG7112 alone or in combination was capable of reducing the *JAK2V617F*^+^ hematopoietic HSPC. CD34^+^ cells were assayed from 6 NDs and 16 different MF patients, among these MF cases, 12 of 16 were *JAK2V617F*^+^ and the remaining 4 were CALR mutation^+^. Treatment with ONC201 or RG7112 alone or in combination did not decrease colony formation by ND CD34^+^ cells (Fig. 6A). By contrast, treatment with ONC201 alone decreased the total number of hematopoietic colonies generated by MF CD34^+^ cells in a dose-dependent fashion. While treatment with ONC201 (10 μM) decreased MF colony numbers by 50%, and RG7112 (500 nM) decreased MF colony numbers by 30%, combination treatment decreased colony numbers by 70% (Fig. 6B). The degree of inhibition of colony numbers observed with each drug alone or combination was similar irrespective of the patient’s with JAK2V617F or CALR mutational status (Suppl. Fig. 6).

To explore the *JAK2* genotype of the MF hematopoietic colonies, individual colonies were randomly plucked from 9 different cases of *JAK2V617F*^+^ MF and then genotyped. Treatment with ONC201 and RG7112 alone decreased the absolute number of *JAK2V617F*^*+*^ colonies, combination treatment with ONC201 + RG7112 decreased *JAK2V617F*^*+*^ colonies to a greater degree than each drug alone in 9 cases (Fig. 6C). Further analysis data showed that ONC201 alone decreased the absolute number of *JAK2V617F*^+^ homozygous colonies by at least 20% in 4 of 9 evaluable cases, and decreased the number of *JAK2V617F*^+^ heterozygous colony numbers by 25% in 6 of 9 cases (Table 1, Fig. 6D). Treatment with RG7112 depleted *JAK2V617F*^+^ homozygous colony numbers in 2 of 9 evaluable cases, and decreased the absolute number of *JAK2V617F*^+^ heterozygous colony number in 7 of 9 cases by 40% (Table 1). The treatment with the ONC201 + RG7112 reduced the total number of both homozygous and heterozygous *JAK2V617F*^+^ colonies to a greater extent than either drug alone in each of 9 cases (Table 1, Fig. 6D, E). Combination treatment more effectively depleted *JAK2V617F*^+^ heterozygous colonies than *JAK2V617F*^*+*^ homozygous colonies (Fig. 6F). Treatment with ONC201 alone led to the appearance of greater numbers of JAK2WT colonies in 2 of 9 cases, while treatment of cells from 1 of the 9 patients with RG7112 resulted in the generation of greater numbers of JAK2WT colonies. Treatment with ONC201 + RG7112, however, increased the absolute numbers of JAK2WT colonies in 4 of 9 cases and allowed the persistence of JAK2WT colonies in 2 of 9 cases albeit at reduced numbers (Table 1).

**Table 1:** Effects of treatment with ONC201 and RG7112 alone or in combination on the absolute numbers of hematopoietic colonies with a specific JAK2 genotype.

	JAK2 Genotype	Control	ONC-10uM	RG-500nM	ONC-RG
Case 1	Hetero^a^	108	65 (60%)^d^	67 (62%)	47 (43%)
Case 2	Homo^b^	94	64 (68%)	86 (91%)	57 (61%)
Case 3	Homo	116	73 (63%)	64 (55%)	27 (23%)
	Hetero	84	63 (75%)	75 (89%)	39 (46%)
	WT^c^	0	0	0	6
Case 4	Homo	60	29 (48%)	60 (100%)	26 (43%)
	Hetero	137	86 (63%)	83 (61%)	37 (27%)
	WT	4	0	0	22
Case 5	Homo	101	104 (103%)	155 (155%)	62 (62%)
	Hetro	139	13 (9.3%)	8 (6%)	7 (5%)
	WT	0	24	0	14
Case 6	Homo	36	35 (100%)	46 (128%)	18 (50%)
	Hetro	53	8 (15%)	13 (25%)	2 (4%)
	WT	0	8	17	14
Case 7	Homo	0	0	0	0
	Hetro	16	13 (81%)	8 (50%)	4 (25%)
	WT	79	62	56	42
Case 8	Homo	10	8 (20%)	7 (30%)	3 (30%)
	Hetro	0	0	0	0
	WT	117	89	106	69
Case 9	Homo	58	74	81	11 (19%)
	Hetro	94	26 (28 %)	24 (26%)	9 (10 %)
	WT	0	0	0	0

Note: a Hetero-Heterozygous, b Homo-homozygous, c WT-Wild type, d percentage of colonies with specific JAK2 genotype that persist after indicated drug treatment.

## Discussion

Ruxolitinib and fedratinib, two competitive JAK2 inhibitors, are the only drugs currently approved to treat MF patients, yet these drugs, although capable of improving symptom burdens, have a modest effect on prolonging patient survival [11–13]. These limitations of JAK2 inhibitor therapy are likely due to the lack of specificity of the presently used JAK2 inhibitors resulting in the inhibition of both mutated and WTJAK2 and the accompanying JAK2 oncogenic activation [11–13, 47]. Furthermore, the majority of MF patients become resistant or intolerant to ruxolitinib therapy after approximately 3 years of use [48]. Thus, the identification of additional novel approaches to treat MF clearly represents an unmet need. It is anticipated that a successful strategy for treating MF that would culminate in a substantial improvement in overall MF patient survival would ultimately require depletion or elimination of malignant HSPCs, the emergence of normal hematopoiesis and be associated with modest hematological and non-hematological toxicity.

We and others previously reported that the progression of MF to more overt phases of the disease and to MPN-BP is associated with abnormalities in the *TP53* pathway including deletion of *TP53*, acquisition of inactivating mutations, and up-regulation of negative regulators of p53 including MDM2/MDM4 and PPM1D [23]. Rampal et al. have also shown in murine models that expression of *JAK2V617F* combined with TP53^−/−^ leads to a fully penetrant form of acute leukemia indicating the importance of the loss of p53 activity in disease progression [49, 50]. Due to these multiple abnormalities in *TP53* observed with MF disease progression, we planned to combine nutlin with ONC201, a drug that activates the extrinsic apoptosis pathway by increasing the extracellular ligand TRAIL expression which involves the binding of extracellular pro-apoptotic ligands to extracellular death receptors, activating downstream signaling to induce apoptosis [31].

Others have reported that the mechanism of action of ONC201 is reliant on tissue-specific pathways underlying the induced stress response, which differs between tumors [51]. Several groups have reported that the effects of ONC201 were not uniformly dependent on either caspase-8 activation or transcription of TRAIL/DR5 but rather due to pathological disturbances that promote the accumulation of unfolded/misfolded proteins, and targeting mitochondrial respiration [35, 52]. Ishizawa and Graves both showed that ONC201 is capable of promoting AML cell lines apoptosis in the absence of caspase activation by activating ClpP, which leads to selective degradation of mitochondrial structure and function as well as degradation of respiratory protein substrates [35–37]. We have demonstrated that MF CD34^+^ cells are characterized by constitutive elevation of ClpP, the situation as regards to the significance of ClpP elevation between AML and MF cells is, however, not entirely analogous. ONC201 alone upregulated ClpP in AML cells which led to the disruption of oxidative phosphorylation and cell death, these effects of ONC201 on AML cells were shown to be independent of TRAIL, and caspases 3 and 8. In this study, although ONC201 alone and RG7112 alone did not further upregulate MF CD34^+^ ClpP transcript levels, ONC201 + RG7112 did increase ClpP transcript levels, we also observed that RG7112 and ONC201 + RG7112 increased ClpP protein levels. It indicates that ClpP is upregulated by p53 activation in MF CD34^+^ cells. RG7112 + ONC201 upregulated ATF4 which has been reported to induce the atypical integrated stress response by ONC201 on AML cells [51]. We also observed that RG7112 alone and RG7112 + ONC201 levels increased the levels of CHOP transcript and protein in MF CD34^+^ cells, which plays a role in mitochondrial-induced unfolded protein response. We observed that treatment with RG7112 alone or in combination with ONC201 elevated the levels of p-EIF2α in MF CD34^+^ cells. These data suggest that treatment with ONC201 + RG7112 induces apoptosis in MF CD34^+^ cells partially though activation of the ER-mitochondrial stress response. Unfortunately, we were unable to evaluate the contribution of ClpP upregulation by ONC201 and RG7112 on MF CD34^+^ cell oxidative phosphorylation due to inadequate access to the needed numbers of primary MF CD34^+^ cells to execute such studies. Ishisawa was however, able to test the effects of ONC201 observed a reduction in respiratory chain proteins in primary AML cells [51]. However, imipridone treatment similarly reduced respiratory chain proteins in normal hematopoietic cells. The preferential effect of ONC201 was attributed to the greater sensitivity of AML cells to ClpP activation due to their increased reliance on oxidative phosphorylation which might not be present in MF CD34^+^ cells [53].

In this study, we have found that TRAIL, DR4, and DR5 were each expressed at lower levels by MF than ND CD34^+^ cells and treatment of MF CD34^+^ cells with ONC201 or RG7112 alone upregulated these components of the extrinsic apoptosis pathway which was further accentuated by treatment with a combination of these drugs leading to activation of cleaved caspases 8/3, in the meantime, we also found that ONC201 enhanced activation the intrinsic apoptosis pathway which was induced by treatment with RG7112 alone or direct activation of *TP53* transcription. These findings indicate the complementary effects of treatment with ONC201 + RG7112 on targeting MF CD34^+^ cells.

We have provided evidence that ONC201 and RG7112 induced apoptosis of MF CD34^+^ cells but not ND CD34^+^ cells and that the actions of these two drugs were additive on MF CD34^+^ cell apoptosis. Importantly, we found that treatment with ONC201 could increase the effects of activation of p53, and further decrease MF CD34^+^ cell numbers. The synergistic effects of combination treatment with ONC201 + RG7112 on apoptosis in MF CD34^+^ cells occurred partially through activation of the p53 pathway. The effects of combination treatment led to a profound reduction in the absolute number of assayable *JAK2V617F*^+^ HSPCs that exceeded the effects of either drug alone. Furthermore, ONC201 + RG7112 was associated with the emergence or persistence of considerable numbers of *JAK2WT* colonies.

**Fig. 7:**
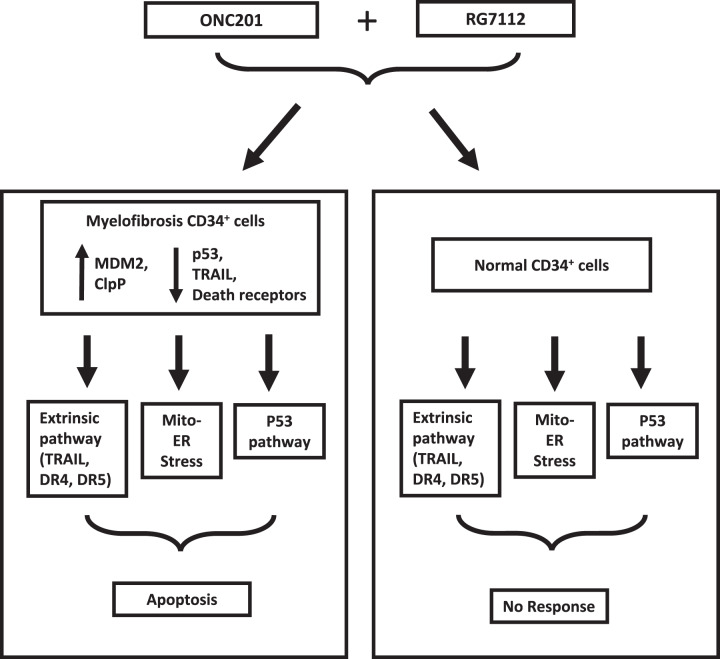
A schema showing that the treatment of MF but not normal CD34^+^ cells with a combination of ONC201 and RG7112 activates both P53-dependent and -independent pathways that leads to apoptosis. MF CD34^+^ cells are characterized by increased levels of MDM2 and ClpP but decreased p53 activity, TRAIL, DR4/5 expression as compared to normal CD34 + cells. These characteristics permit ONC201 + RG7112 to selectively deplete MF HSC/HPCs.

In conclusion, this study indicates that ONC201 is a potentially effective drug for the treatment of MF. The combination of ONC201 + RG7112 selectively targets MF CD34^+^ cells by inducing apoptosis through both intrinsic and extrinsic apoptosis pathways (Fig. 7). The ability of this drug combination to spare normal CD34^+^ cells might allow it to be better tolerated in MF patients that have an associated marrow failure state. Such a therapeutic approach would be anticipated to promote the elimination of both *TP53*-wild type and *TP53*-mutated MF HSPCs resulting in the depletion of MF but not normal HSPCs and further be issued a clinical trial in MF.

## Supplementary information

Combined Drug Targeting of p53-dependent and -independent Pathways Depletes Myelofibrosis Hematopoietic Stem/Progenitor Cells
