# Supplementary material for: Combined Drug Targeting of p53-dependent and -independent Pathways Depletes Myelofibrosis Hematopoietic Stem/Progenitor Cells
Source: Leukemia. 2021 Oct 12;36(3):733–45. doi: 10.1038/s41375-021-01446-4 (PMC8885407; doi:10.1038/s41375-021-01446-4)
Supplement: Supplementary file 1 — Combined Drug Targeting of p53-dependent and -independent Pathways Depletes Myelofibrosis Hematopoietic Stem/Progenitor Cells [file 41375_2021_1446_MOESM1_ESM.pptx]

## Slide 1
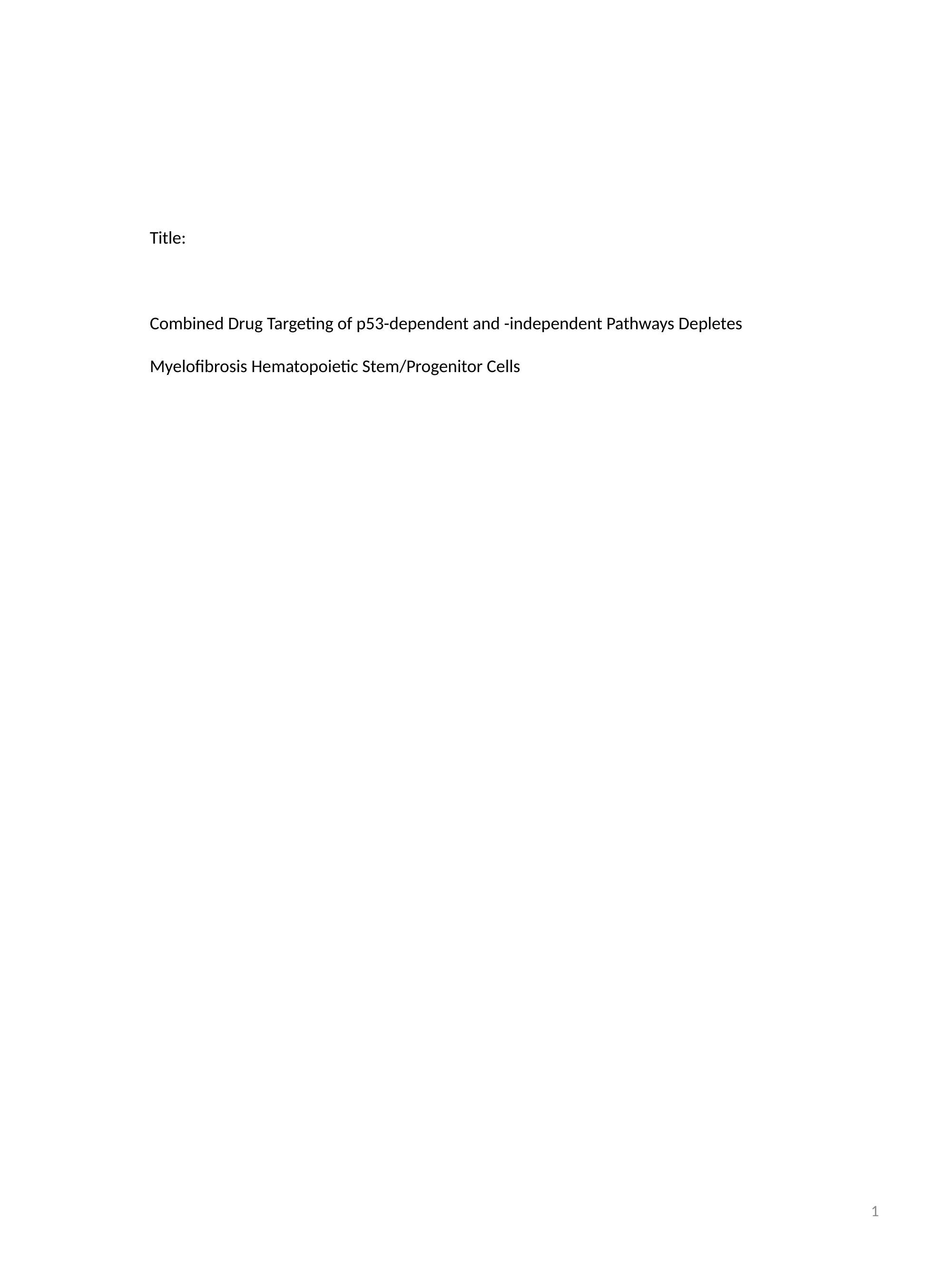

Title:
Combined Drug Targeting of p53-dependent and -independent Pathways Depletes Myelofibrosis Hematopoietic Stem/Progenitor Cells
1

## Slide 2
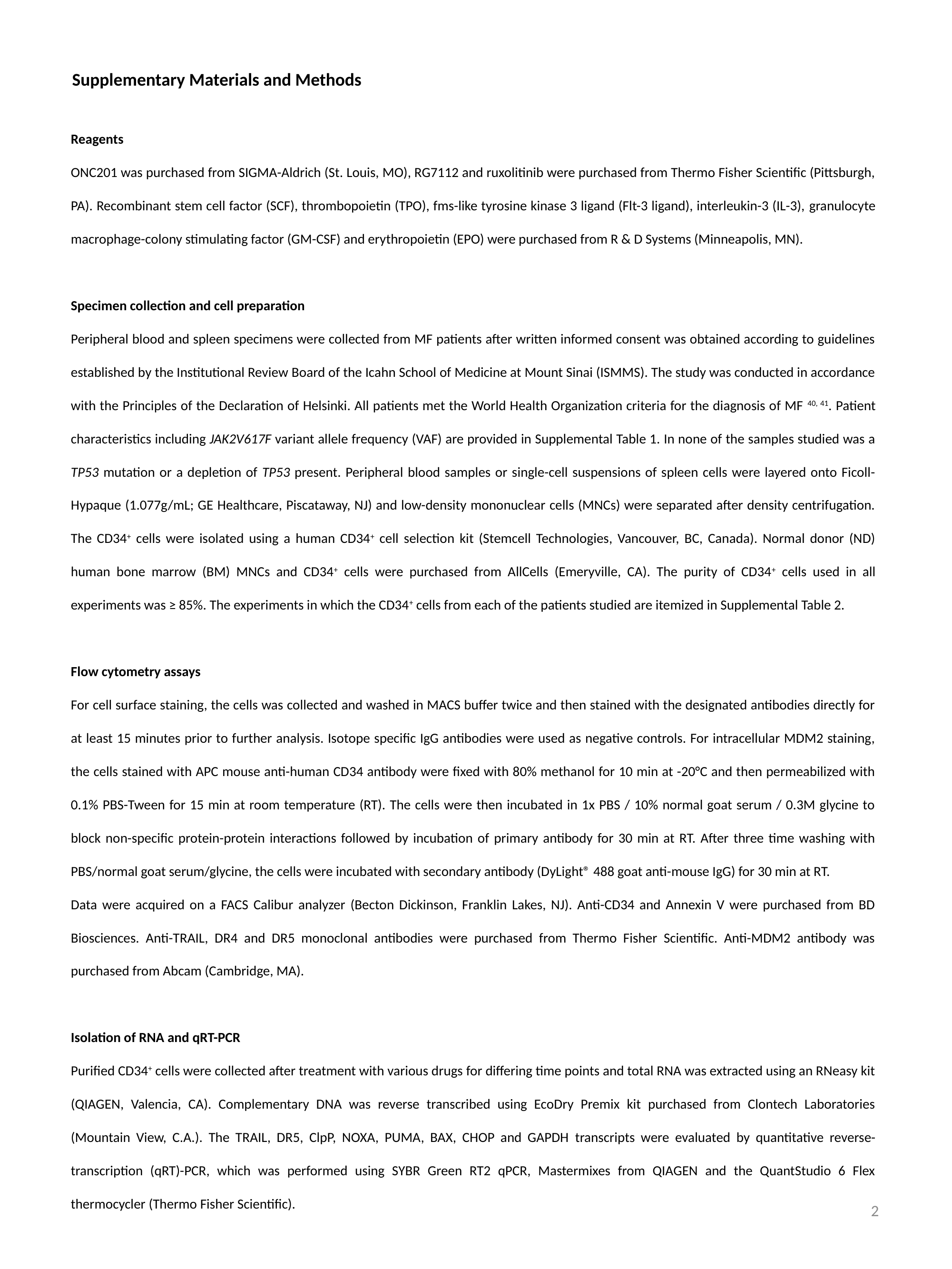

Supplementary Materials and Methods
Reagents
ONC201 was purchased from SIGMA-Aldrich (St. Louis, MO), RG7112 and ruxolitinib were purchased from Thermo Fisher Scientific (Pittsburgh, PA). Recombinant stem cell factor (SCF), thrombopoietin (TPO), fms-like tyrosine kinase 3 ligand (Flt-3 ligand), interleukin-3 (IL-3), granulocyte macrophage-colony stimulating factor (GM-CSF) and erythropoietin (EPO) were purchased from R & D Systems (Minneapolis, MN).
Specimen collection and cell preparation
Peripheral blood and spleen specimens were collected from MF patients after written informed consent was obtained according to guidelines established by the Institutional Review Board of the Icahn School of Medicine at Mount Sinai (ISMMS). The study was conducted in accordance with the Principles of the Declaration of Helsinki. All patients met the World Health Organization criteria for the diagnosis of MF 40, 41. Patient characteristics including JAK2V617F variant allele frequency (VAF) are provided in Supplemental Table 1. In none of the samples studied was a TP53 mutation or a depletion of TP53 present. Peripheral blood samples or single-cell suspensions of spleen cells were layered onto Ficoll-Hypaque (1.077g/mL; GE Healthcare, Piscataway, NJ) and low-density mononuclear cells (MNCs) were separated after density centrifugation. The CD34+ cells were isolated using a human CD34+ cell selection kit (Stemcell Technologies, Vancouver, BC, Canada). Normal donor (ND) human bone marrow (BM) MNCs and CD34+ cells were purchased from AllCells (Emeryville, CA). The purity of CD34+ cells used in all experiments was ≥ 85%. The experiments in which the CD34+ cells from each of the patients studied are itemized in Supplemental Table 2.
Flow cytometry assays
For cell surface staining, the cells was collected and washed in MACS buffer twice and then stained with the designated antibodies directly for at least 15 minutes prior to further analysis. Isotope specific IgG antibodies were used as negative controls. For intracellular MDM2 staining, the cells stained with APC mouse anti-human CD34 antibody were fixed with 80% methanol for 10 min at -20°C and then permeabilized with 0.1% PBS-Tween for 15 min at room temperature (RT). The cells were then incubated in 1x PBS / 10% normal goat serum / 0.3M glycine to block non-specific protein-protein interactions followed by incubation of primary antibody for 30 min at RT. After three time washing with PBS/normal goat serum/glycine, the cells were incubated with secondary antibody (DyLight® 488 goat anti-mouse IgG) for 30 min at RT.
Data were acquired on a FACS Calibur analyzer (Becton Dickinson, Franklin Lakes, NJ). Anti-CD34 and Annexin V were purchased from BD Biosciences. Anti-TRAIL, DR4 and DR5 monoclonal antibodies were purchased from Thermo Fisher Scientific. Anti-MDM2 antibody was purchased from Abcam (Cambridge, MA).
Isolation of RNA and qRT-PCR
Purified CD34+ cells were collected after treatment with various drugs for differing time points and total RNA was extracted using an RNeasy kit (QIAGEN, Valencia, CA). Complementary DNA was reverse transcribed using EcoDry Premix kit purchased from Clontech Laboratories (Mountain View, C.A.). The TRAIL, DR5, ClpP, NOXA, PUMA, BAX, CHOP and GAPDH transcripts were evaluated by quantitative reverse-transcription (qRT)-PCR, which was performed using SYBR Green RT2 qPCR, Mastermixes from QIAGEN and the QuantStudio 6 Flex thermocycler (Thermo Fisher Scientific).
2

## Slide 3
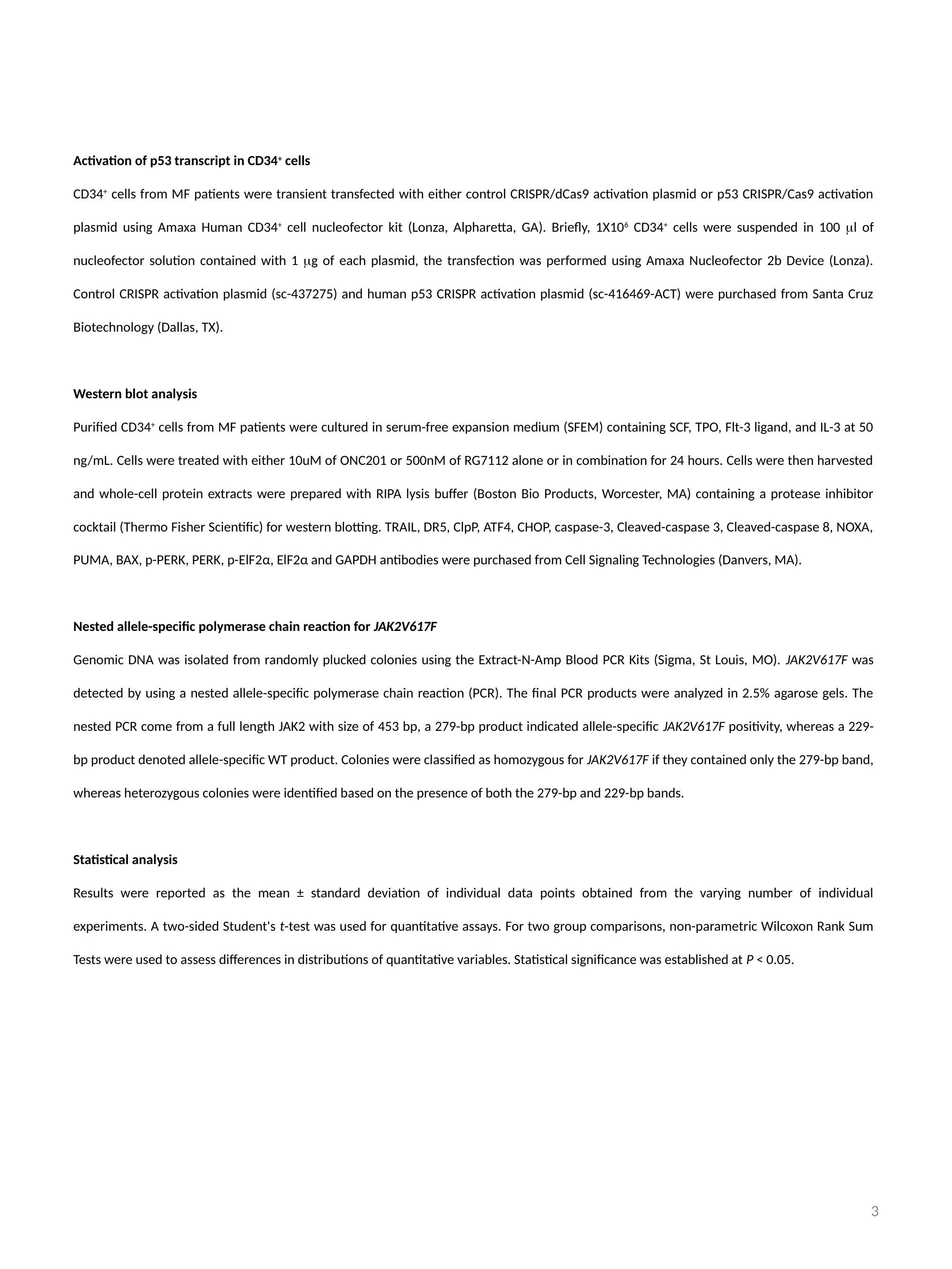

Activation of p53 transcript in CD34+ cells
CD34+ cells from MF patients were transient transfected with either control CRISPR/dCas9 activation plasmid or p53 CRISPR/Cas9 activation plasmid using Amaxa Human CD34+ cell nucleofector kit (Lonza, Alpharetta, GA). Briefly, 1X106 CD34+ cells were suspended in 100 μl of nucleofector solution contained with 1 μg of each plasmid, the transfection was performed using Amaxa Nucleofector 2b Device (Lonza). Control CRISPR activation plasmid (sc-437275) and human p53 CRISPR activation plasmid (sc-416469-ACT) were purchased from Santa Cruz Biotechnology (Dallas, TX).
Western blot analysis
Purified CD34+ cells from MF patients were cultured in serum-free expansion medium (SFEM) containing SCF, TPO, Flt-3 ligand, and IL-3 at 50 ng/mL. Cells were treated with either 10uM of ONC201 or 500nM of RG7112 alone or in combination for 24 hours. Cells were then harvested and whole-cell protein extracts were prepared with RIPA lysis buffer (Boston Bio Products, Worcester, MA) containing a protease inhibitor cocktail (Thermo Fisher Scientific) for western blotting. TRAIL, DR5, ClpP, ATF4, CHOP, caspase-3, Cleaved-caspase 3, Cleaved-caspase 8, NOXA, PUMA, BAX, p-PERK, PERK, p-ElF2α, ElF2α and GAPDH antibodies were purchased from Cell Signaling Technologies (Danvers, MA).
Nested allele-specific polymerase chain reaction for JAK2V617F
Genomic DNA was isolated from randomly plucked colonies using the Extract-N-Amp Blood PCR Kits (Sigma, St Louis, MO). JAK2V617F was detected by using a nested allele-specific polymerase chain reaction (PCR). The final PCR products were analyzed in 2.5% agarose gels. The nested PCR come from a full length JAK2 with size of 453 bp, a 279-bp product indicated allele-specific JAK2V617F positivity, whereas a 229- bp product denoted allele-specific WT product. Colonies were classified as homozygous for JAK2V617F if they contained only the 279-bp band, whereas heterozygous colonies were identified based on the presence of both the 279-bp and 229-bp bands.
Statistical analysis
Results were reported as the mean ± standard deviation of individual data points obtained from the varying number of individual experiments. A two-sided Student's t-test was used for quantitative assays. For two group comparisons, non-parametric Wilcoxon Rank Sum Tests were used to assess differences in distributions of quantitative variables. Statistical significance was established at P < 0.05.
3

## Slide 4
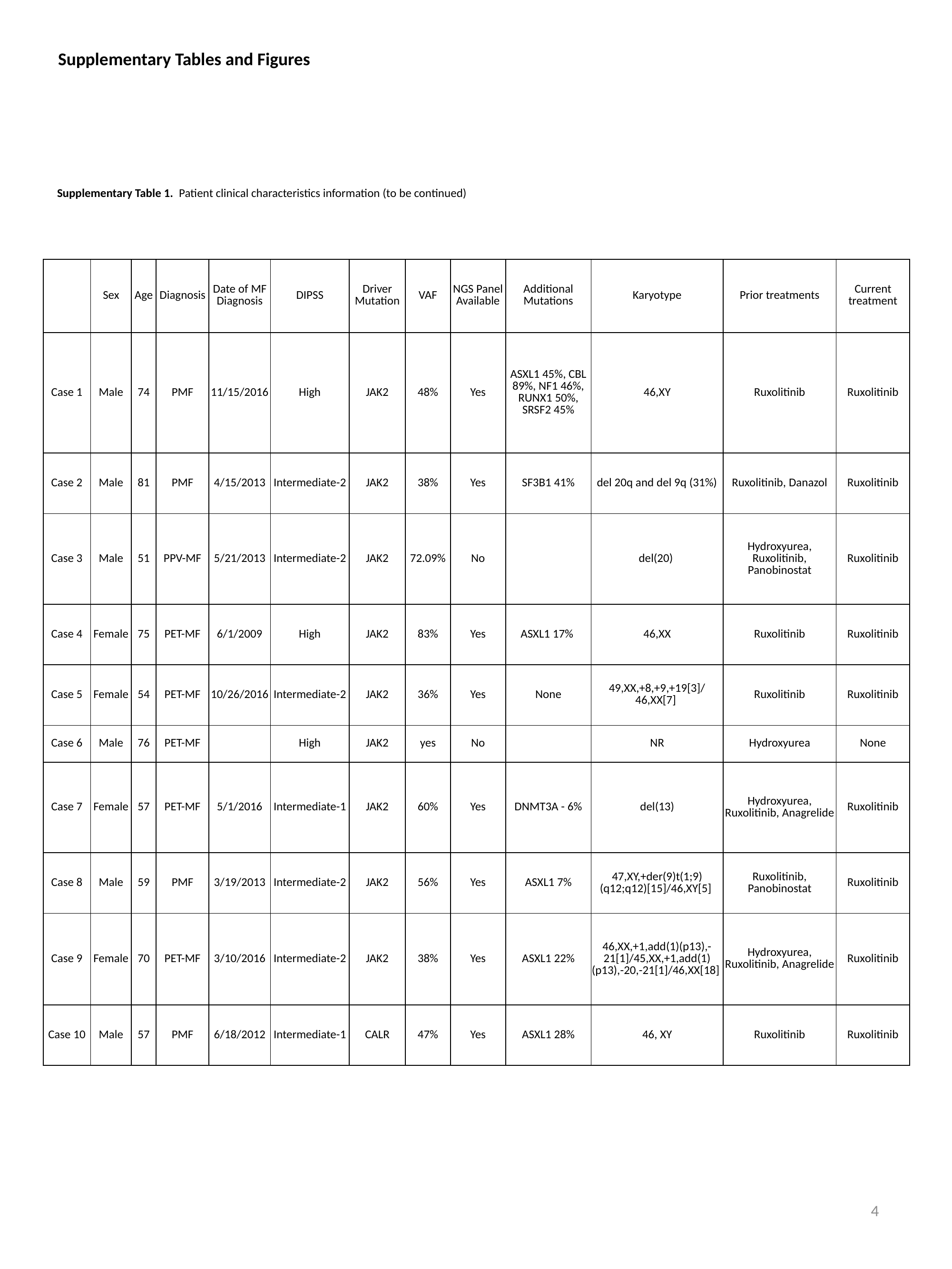

Supplementary Tables and Figures
Supplementary Table 1. Patient clinical characteristics information (to be continued)
| | Sex | Age | Diagnosis | Date of MF Diagnosis | DIPSS | Driver Mutation | VAF | NGS Panel Available | Additional Mutations | Karyotype | Prior treatments | Current treatment |
| --- | --- | --- | --- | --- | --- | --- | --- | --- | --- | --- | --- | --- |
| Case 1 | Male | 74 | PMF | 11/15/2016 | High | JAK2 | 48% | Yes | ASXL1 45%, CBL 89%, NF1 46%, RUNX1 50%, SRSF2 45% | 46,XY | Ruxolitinib | Ruxolitinib |
| Case 2 | Male | 81 | PMF | 4/15/2013 | Intermediate-2 | JAK2 | 38% | Yes | SF3B1 41% | del 20q and del 9q (31%) | Ruxolitinib, Danazol | Ruxolitinib |
| Case 3 | Male | 51 | PPV-MF | 5/21/2013 | Intermediate-2 | JAK2 | 72.09% | No | | del(20) | Hydroxyurea, Ruxolitinib, Panobinostat | Ruxolitinib |
| Case 4 | Female | 75 | PET-MF | 6/1/2009 | High | JAK2 | 83% | Yes | ASXL1 17% | 46,XX | Ruxolitinib | Ruxolitinib |
| Case 5 | Female | 54 | PET-MF | 10/26/2016 | Intermediate-2 | JAK2 | 36% | Yes | None | 49,XX,+8,+9,+19[3]/46,XX[7] | Ruxolitinib | Ruxolitinib |
| Case 6 | Male | 76 | PET-MF | | High | JAK2 | yes | No | | NR | Hydroxyurea | None |
| Case 7 | Female | 57 | PET-MF | 5/1/2016 | Intermediate-1 | JAK2 | 60% | Yes | DNMT3A - 6% | del(13) | Hydroxyurea, Ruxolitinib, Anagrelide | Ruxolitinib |
| Case 8 | Male | 59 | PMF | 3/19/2013 | Intermediate-2 | JAK2 | 56% | Yes | ASXL1 7% | 47,XY,+der(9)t(1;9)(q12;q12)[15]/46,XY[5] | Ruxolitinib, Panobinostat | Ruxolitinib |
| Case 9 | Female | 70 | PET-MF | 3/10/2016 | Intermediate-2 | JAK2 | 38% | Yes | ASXL1 22% | 46,XX,+1,add(1)(p13),-21[1]/45,XX,+1,add(1)(p13),-20,-21[1]/46,XX[18] | Hydroxyurea, Ruxolitinib, Anagrelide | Ruxolitinib |
| Case 10 | Male | 57 | PMF | 6/18/2012 | Intermediate-1 | CALR | 47% | Yes | ASXL1 28% | 46, XY | Ruxolitinib | Ruxolitinib |
4

## Slide 5
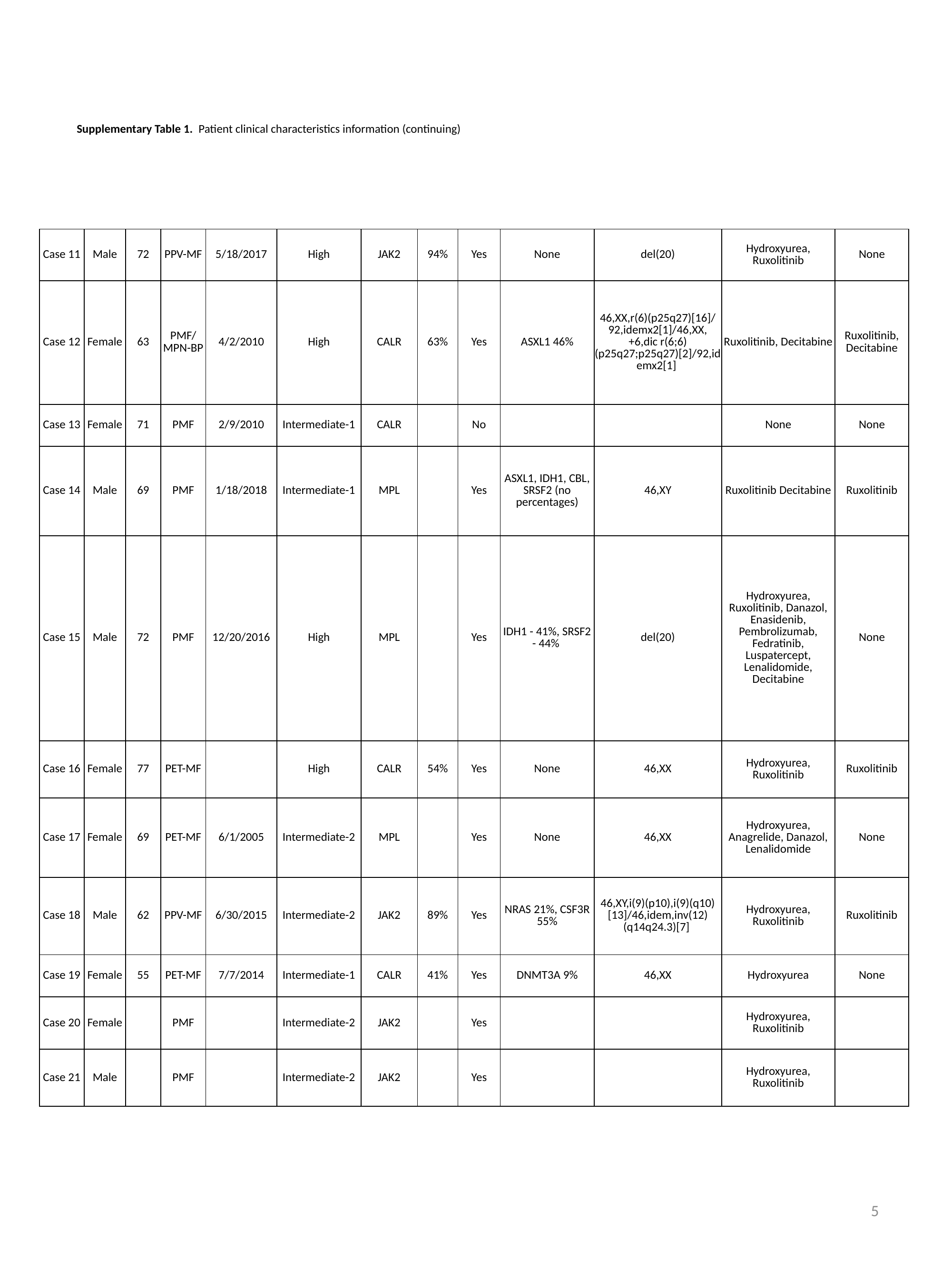

Supplementary Table 1. Patient clinical characteristics information (continuing)
| Case 11 | Male | 72 | PPV-MF | 5/18/2017 | High | JAK2 | 94% | Yes | None | del(20) | Hydroxyurea, Ruxolitinib | None |
| --- | --- | --- | --- | --- | --- | --- | --- | --- | --- | --- | --- | --- |
| Case 12 | Female | 63 | PMF/MPN-BP | 4/2/2010 | High | CALR | 63% | Yes | ASXL1 46% | 46,XX,r(6)(p25q27)[16]/92,idemx2[1]/46,XX,+6,dic r(6;6)(p25q27;p25q27)[2]/92,idemx2[1] | Ruxolitinib, Decitabine | Ruxolitinib, Decitabine |
| Case 13 | Female | 71 | PMF | 2/9/2010 | Intermediate-1 | CALR | | No | | | None | None |
| Case 14 | Male | 69 | PMF | 1/18/2018 | Intermediate-1 | MPL | | Yes | ASXL1, IDH1, CBL, SRSF2 (no percentages) | 46,XY | Ruxolitinib Decitabine | Ruxolitinib |
| Case 15 | Male | 72 | PMF | 12/20/2016 | High | MPL | | Yes | IDH1 - 41%, SRSF2 - 44% | del(20) | Hydroxyurea, Ruxolitinib, Danazol, Enasidenib, Pembrolizumab, Fedratinib, Luspatercept, Lenalidomide, Decitabine | None |
| Case 16 | Female | 77 | PET-MF | | High | CALR | 54% | Yes | None | 46,XX | Hydroxyurea, Ruxolitinib | Ruxolitinib |
| Case 17 | Female | 69 | PET-MF | 6/1/2005 | Intermediate-2 | MPL | | Yes | None | 46,XX | Hydroxyurea, Anagrelide, Danazol, Lenalidomide | None |
| Case 18 | Male | 62 | PPV-MF | 6/30/2015 | Intermediate-2 | JAK2 | 89% | Yes | NRAS 21%, CSF3R 55% | 46,XY,i(9)(p10),i(9)(q10)[13]/46,idem,inv(12)(q14q24.3)[7] | Hydroxyurea, Ruxolitinib | Ruxolitinib |
| Case 19 | Female | 55 | PET-MF | 7/7/2014 | Intermediate-1 | CALR | 41% | Yes | DNMT3A 9% | 46,XX | Hydroxyurea | None |
| Case 20 | Female | | PMF | | Intermediate-2 | JAK2 | | Yes | | | Hydroxyurea, Ruxolitinib | |
| Case 21 | Male | | PMF | | Intermediate-2 | JAK2 | | Yes | | | Hydroxyurea, Ruxolitinib | |
5

## Slide 6
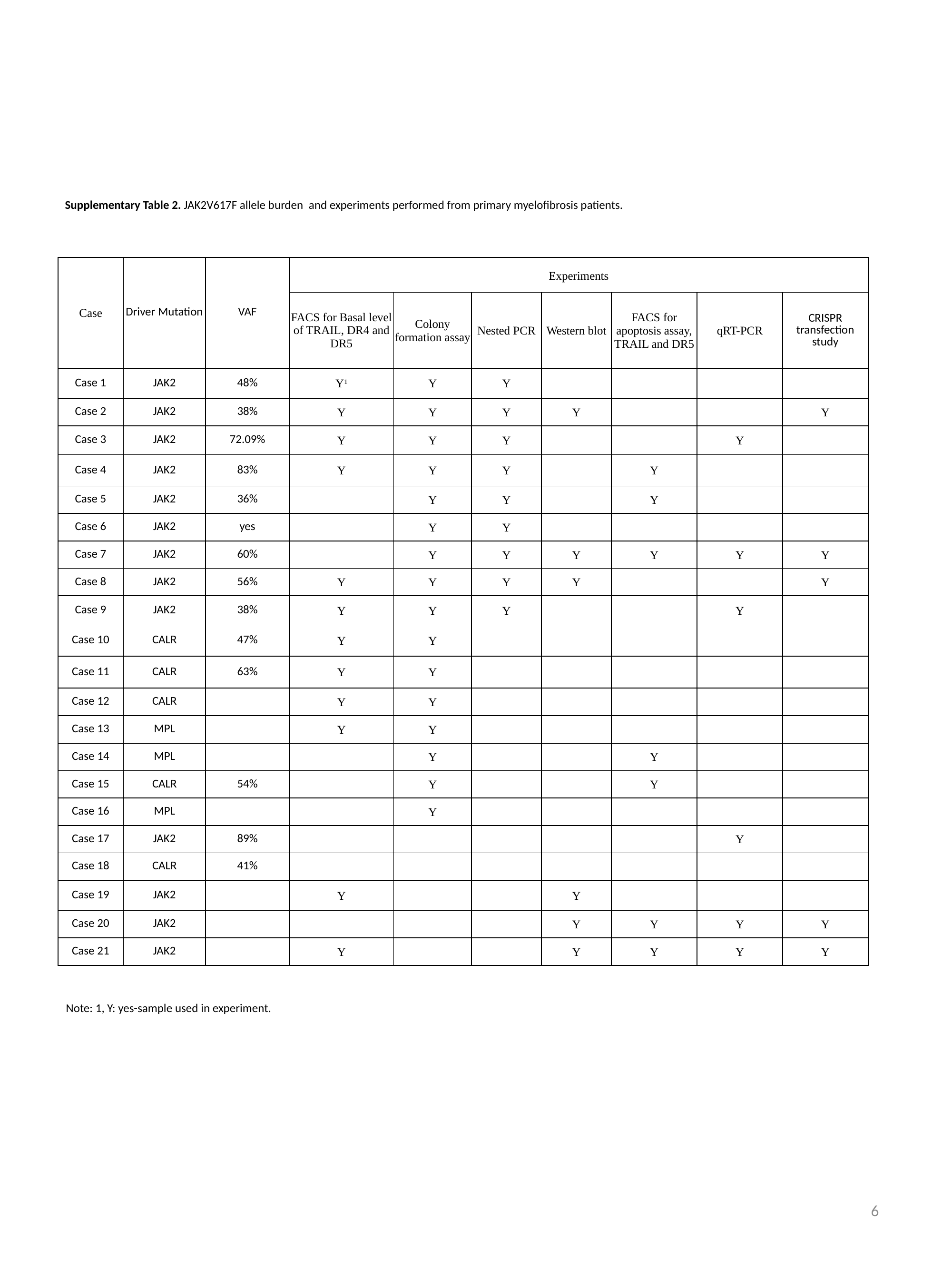

Supplementary Table 2. JAK2V617F allele burden and experiments performed from primary myelofibrosis patients.
| Case | Driver Mutation | VAF | Experiments | | | | | | |
| --- | --- | --- | --- | --- | --- | --- | --- | --- | --- |
| | | | FACS for Basal level of TRAIL, DR4 and DR5 | Colony formation assay | Nested PCR | Western blot | FACS for apoptosis assay, TRAIL and DR5 | qRT-PCR | CRISPR transfection study |
| Case 1 | JAK2 | 48% | Y1 | Y | Y | | | | |
| Case 2 | JAK2 | 38% | Y | Y | Y | Y | | | Y |
| Case 3 | JAK2 | 72.09% | Y | Y | Y | | | Y | |
| Case 4 | JAK2 | 83% | Y | Y | Y | | Y | | |
| Case 5 | JAK2 | 36% | | Y | Y | | Y | | |
| Case 6 | JAK2 | yes | | Y | Y | | | | |
| Case 7 | JAK2 | 60% | | Y | Y | Y | Y | Y | Y |
| Case 8 | JAK2 | 56% | Y | Y | Y | Y | | | Y |
| Case 9 | JAK2 | 38% | Y | Y | Y | | | Y | |
| Case 10 | CALR | 47% | Y | Y | | | | | |
| Case 11 | CALR | 63% | Y | Y | | | | | |
| Case 12 | CALR | | Y | Y | | | | | |
| Case 13 | MPL | | Y | Y | | | | | |
| Case 14 | MPL | | | Y | | | Y | | |
| Case 15 | CALR | 54% | | Y | | | Y | | |
| Case 16 | MPL | | | Y | | | | | |
| Case 17 | JAK2 | 89% | | | | | | Y | |
| Case 18 | CALR | 41% | | | | | | | |
| Case 19 | JAK2 | | Y | | | Y | | | |
| Case 20 | JAK2 | | | | | Y | Y | Y | Y |
| Case 21 | JAK2 | | Y | | | Y | Y | Y | Y |
Note: 1, Y: yes-sample used in experiment.
6

## Slide 7
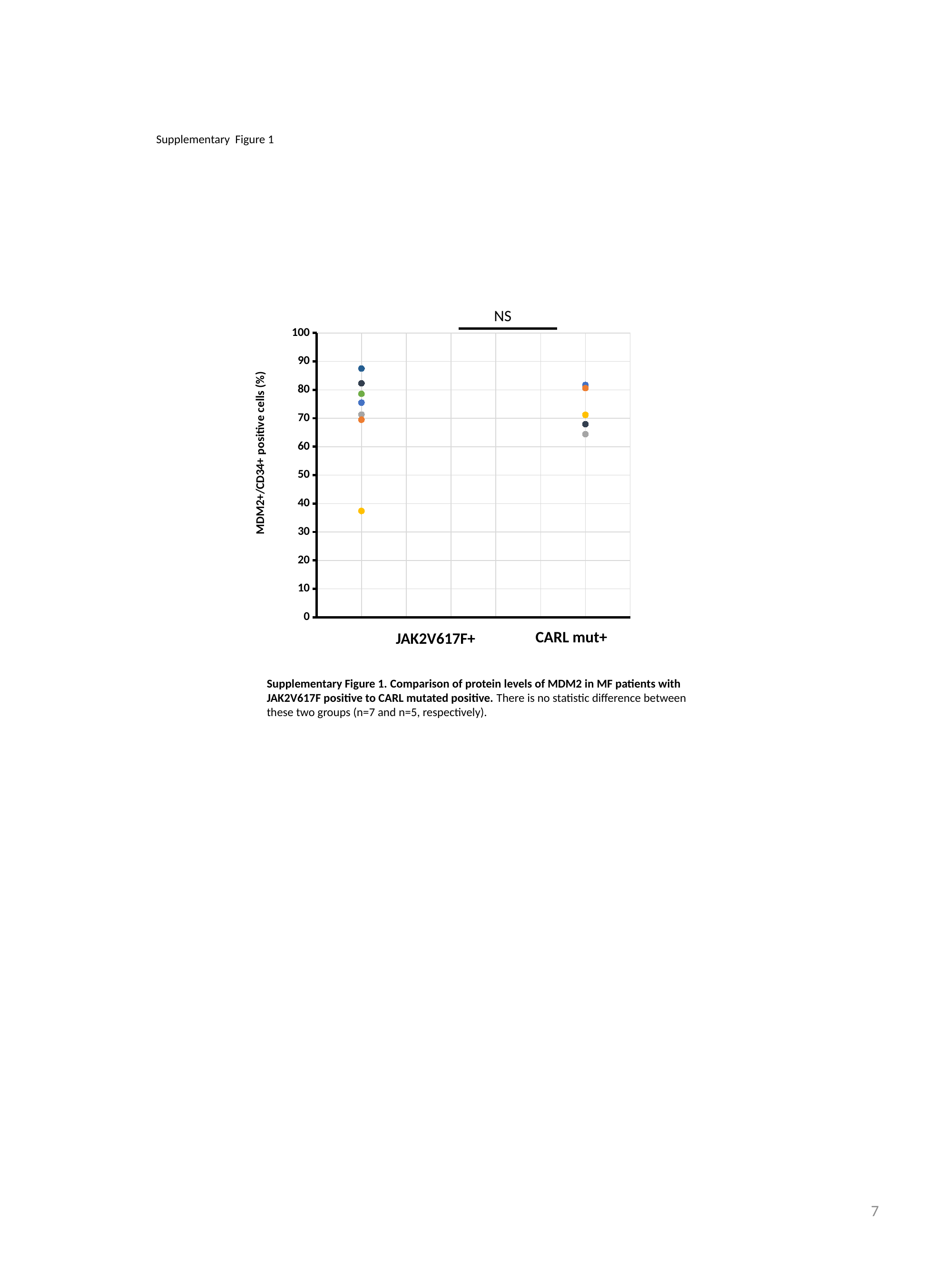

Supplementary Figure 1
NS
### Chart
| Category | | | | | | | |
|---|---|---|---|---|---|---|---|CARL mut+
JAK2V617F+
Supplementary Figure 1. Comparison of protein levels of MDM2 in MF patients with JAK2V617F positive to CARL mutated positive. There is no statistic difference between these two groups (n=7 and n=5, respectively).
7

## Slide 8
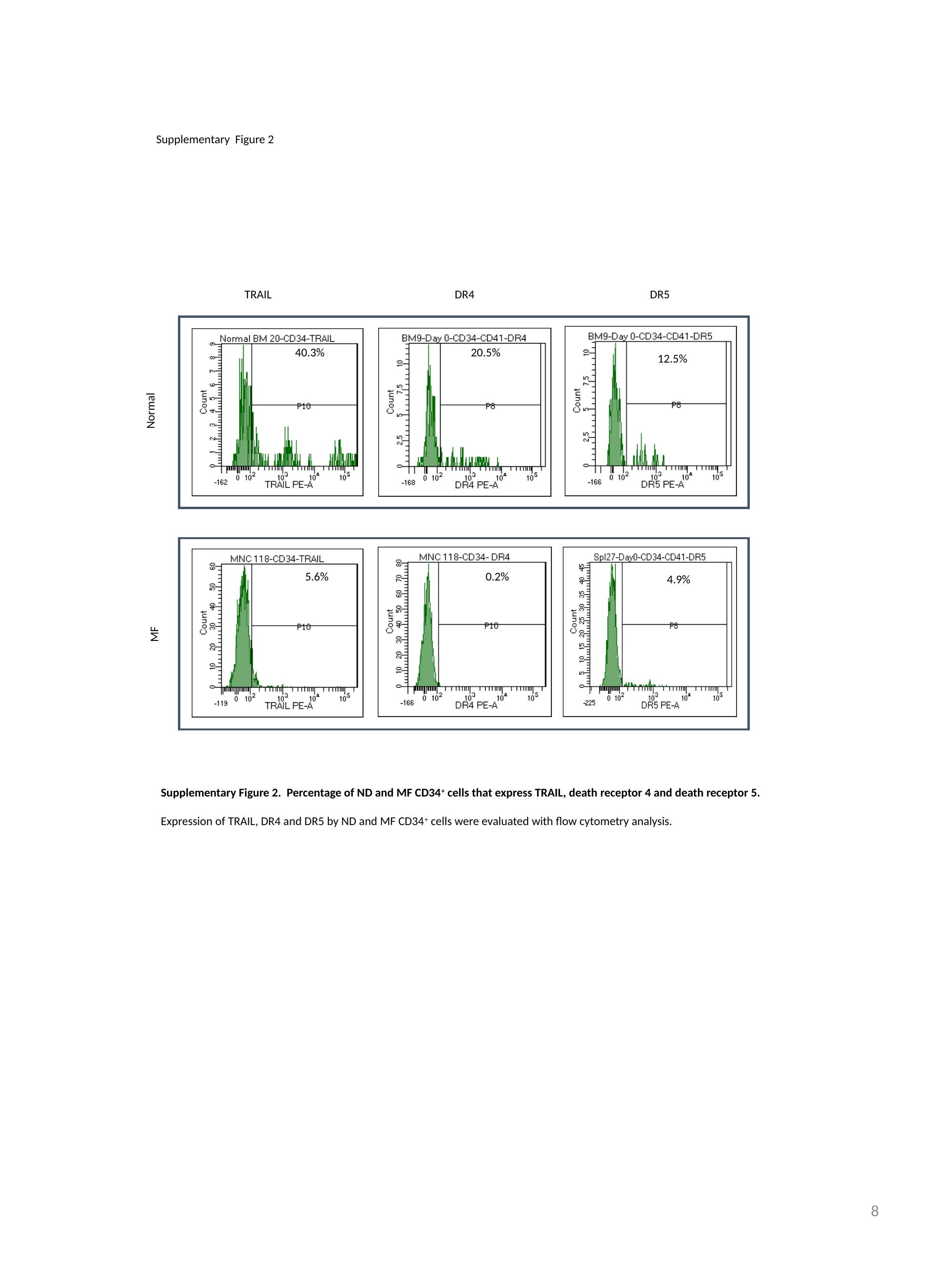

Supplementary Figure 2
TRAIL
DR4
DR5
40.3%
20.5%
12.5%
Normal
0.2%
5.6%
4.9%
MF
Supplementary Figure 2. Percentage of ND and MF CD34+ cells that express TRAIL, death receptor 4 and death receptor 5. Expression of TRAIL, DR4 and DR5 by ND and MF CD34+ cells were evaluated with flow cytometry analysis.
8

## Slide 9
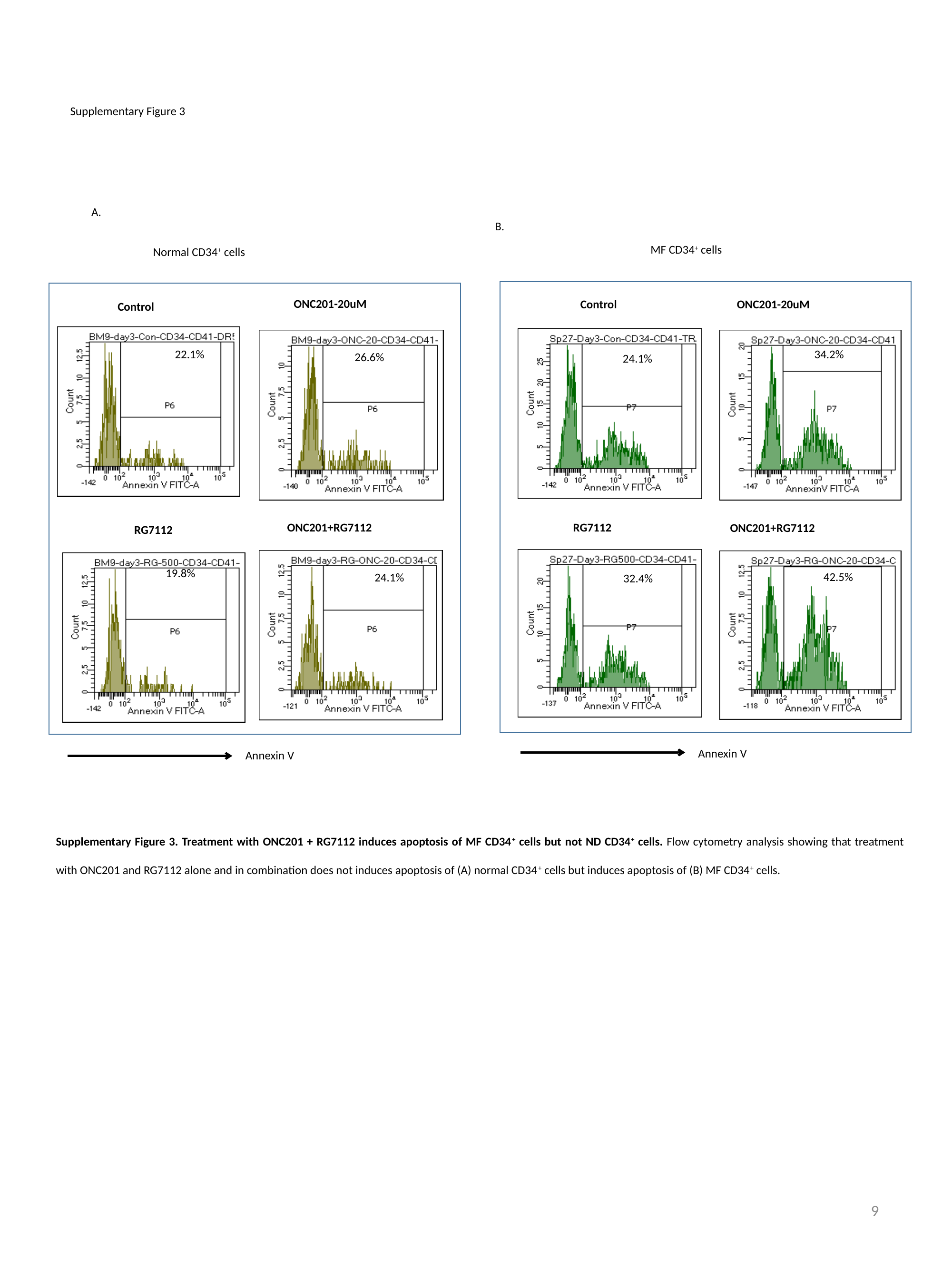

Supplementary Figure 3
A.
B.
MF CD34+ cells
Normal CD34+ cells
ONC201-20uM
Control
ONC201-20uM
Control
22.1%
24.1%
34.2%
26.6%
ONC201+RG7112
RG7112
ONC201+RG7112
RG7112
32.4%
24.1%
42.5%
19.8%
Annexin V
Annexin V
Supplementary Figure 3. Treatment with ONC201 + RG7112 induces apoptosis of MF CD34+ cells but not ND CD34+ cells. Flow cytometry analysis showing that treatment with ONC201 and RG7112 alone and in combination does not induces apoptosis of (A) normal CD34+ cells but induces apoptosis of (B) MF CD34+ cells.
9

## Slide 10
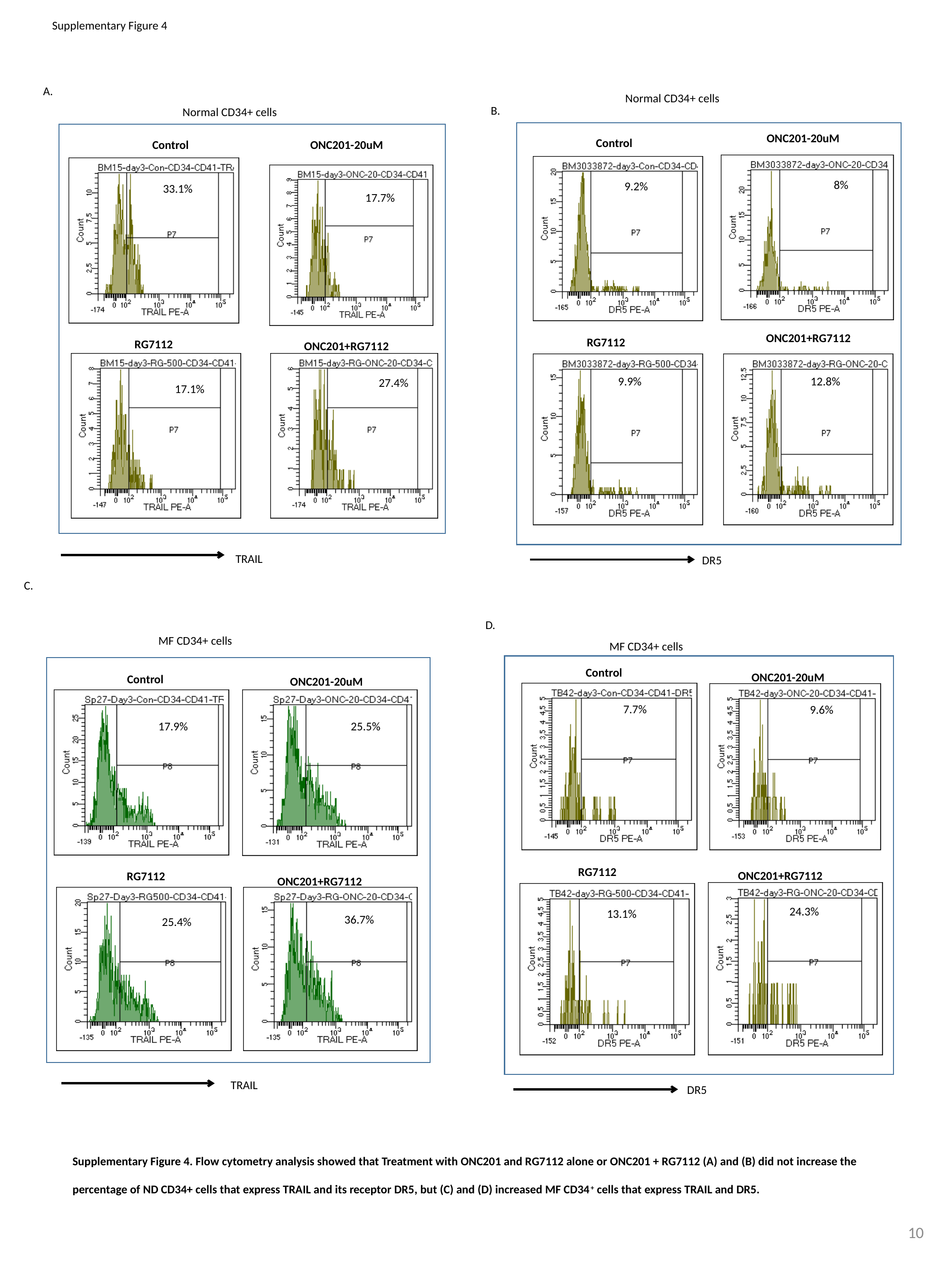

Supplementary Figure 4
A.
Normal CD34+ cells
ONC201-20uM
Control
8%
9.2%
ONC201+RG7112
RG7112
12.8%
9.9%
DR5
B.
Normal CD34+ cells
ONC201-20uM
Control
33.1%
17.7%
RG7112
ONC201+RG7112
17.1%
27.4%
TRAIL
C.
D.
MF CD34+ cells
Control
ONC201-20uM
17.9%
25.5%
RG7112
ONC201+RG7112
36.7%
25.4%
TRAIL
MF CD34+ cells
Control
ONC201-20uM
7.7%
9.6%
RG7112
ONC201+RG7112
24.3%
13.1%
DR5
Supplementary Figure 4. Flow cytometry analysis showed that Treatment with ONC201 and RG7112 alone or ONC201 + RG7112 (A) and (B) did not increase the percentage of ND CD34+ cells that express TRAIL and its receptor DR5, but (C) and (D) increased MF CD34+ cells that express TRAIL and DR5.
10

## Slide 11
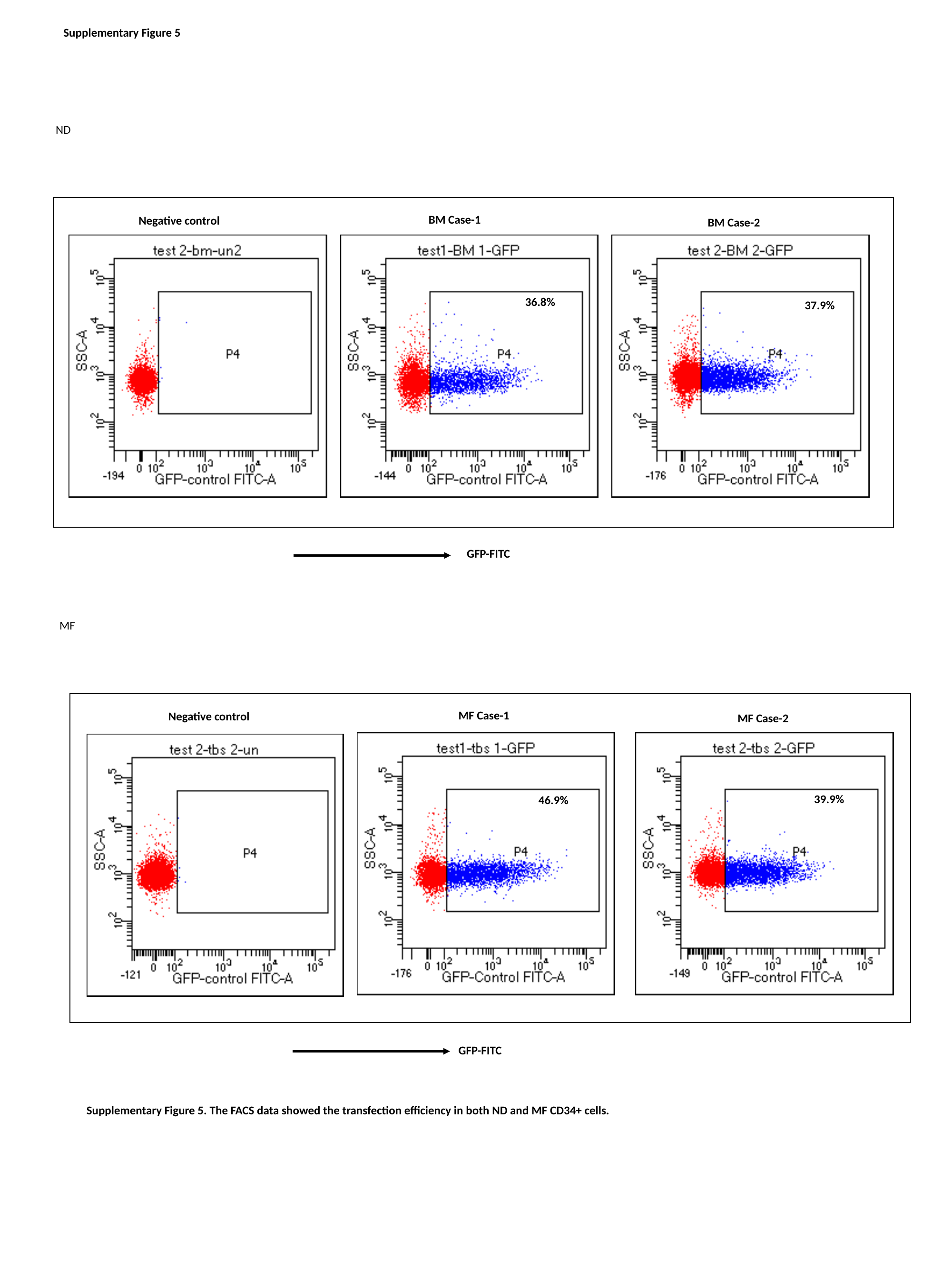

Supplementary Figure 5
ND
36.8%
37.9%
BM Case-1
Negative control
BM Case-2
GFP-FITC
MF
46.9%
39.9%
MF Case-1
Negative control
MF Case-2
GFP-FITC
Supplementary Figure 5. The FACS data showed the transfection efficiency in both ND and MF CD34+ cells.

## Slide 12
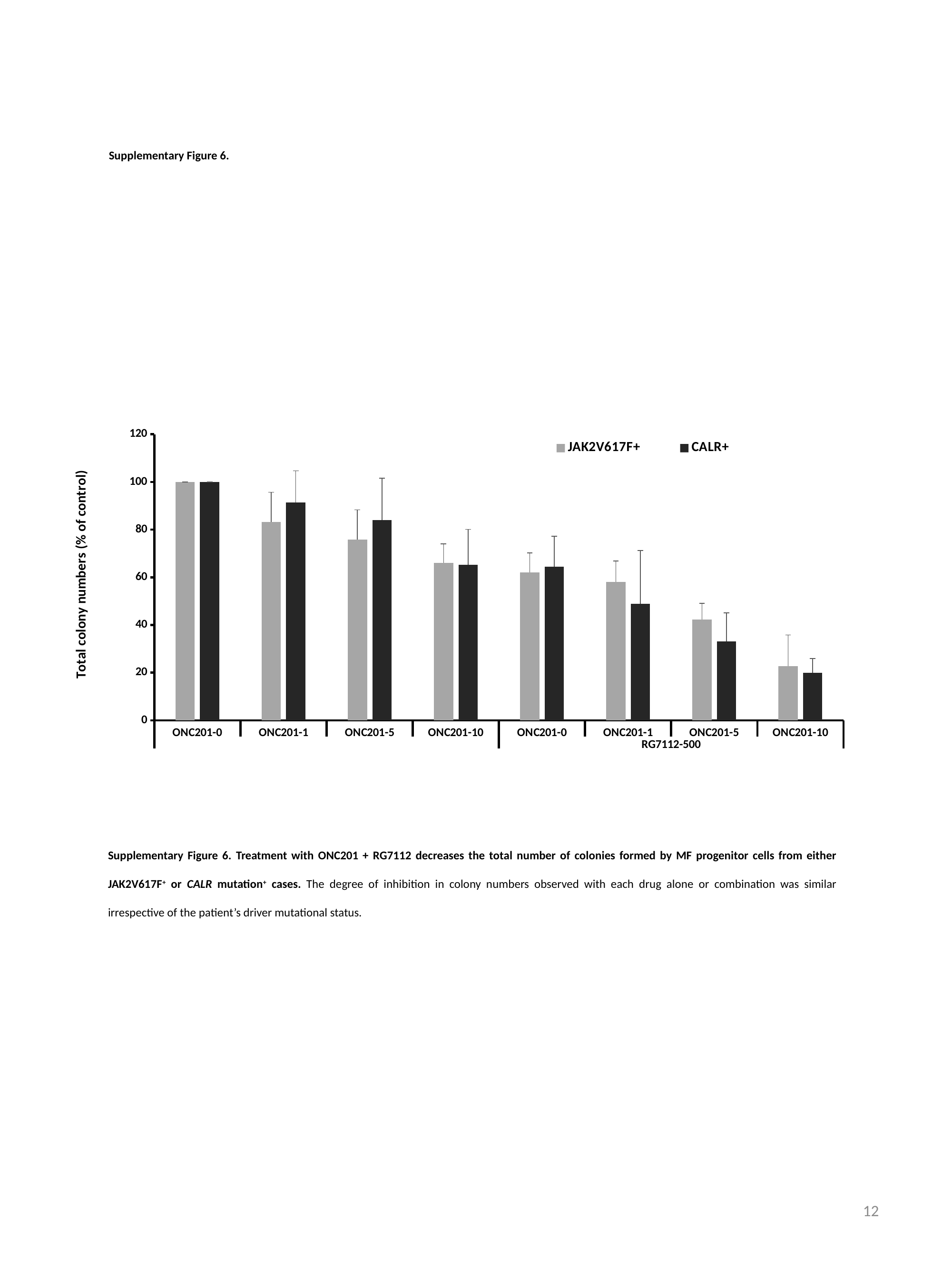

Supplementary Figure 6.
### Chart
| Category | JAK2V617F+ | CALR+ |
|---|---|---|
| ONC201-0 | 100.0 | 100.0 |
| ONC201-1 | 83.13793163645776 | 91.34653036493049 |
| ONC201-5 | 75.86004749752348 | 84.06725249787253 |
| ONC201-10 | 65.96096828392604 | 65.19539370678567 |
| ONC201-0 | 61.967157603957894 | 64.44246230771472 |
| ONC201-1 | 58.01637790186728 | 48.85491677717384 |
| ONC201-5 | 42.365161601100425 | 33.10040884026715 |
| ONC201-10 | 22.837243566759952 | 19.982661929020935 |Supplementary Figure 6. Treatment with ONC201 + RG7112 decreases the total number of colonies formed by MF progenitor cells from either JAK2V617F+ or CALR mutation+ cases. The degree of inhibition in colony numbers observed with each drug alone or combination was similar irrespective of the patient’s driver mutational status.
12
